# Sex-disaggregated outcomes of adverse events after COVID-19 vaccination: A Dutch cohort study and review of the literature

**DOI:** 10.3389/fimmu.2023.1078736

**Published:** 2023-01-30

**Authors:** Janneke W. Duijster, Thomas Lieber, Silvia Pacelli, Leontine Van Balveren, Loes S. Ruijs, Monika Raethke, Agnes Kant, Florence Van Hunsel

**Affiliations:** ^1^ Netherlands Pharmacovigilance Centre Lareb, ‘s-Hertogenbosch, Netherlands; ^2^ School of Pharmacy, Biotechnology, and Sport Sciences, University of Bologna, Bologna, Italy

**Keywords:** COVID-19 vaccine, sex, adverse event after vaccination, patient reported outcome, pharmacovigilance, longitudinal cohort design

## Abstract

**Background:**

Albeit the need for sex-disaggregated results of adverse events after immunization (AEFIs) is gaining attention since the COVID-19 pandemic, studies with emphasis on sexual dimorphism in response to COVID-19 vaccination are relatively scarce. This prospective cohort study aimed to assess differences in the incidence and course of reported AEFIs after COVID-19 vaccination between males and females in the Netherlands and provides a summary of sex-disaggregated outcomes in published literature.

**Methods:**

Patient reported outcomes of AEFIs over a six month period following the first vaccination with BioNTech-Pfizer, AstraZeneca, Moderna or the Johnson&Johnson vaccine were collected in a Cohort Event Monitoring study. Logistic regression was used to assess differences in incidence of ‘any AEFI’, local reactions and the top ten most reported AEFIs between the sexes. Effects of age, vaccine brand, comorbidities, prior COVID-19 infection and the use of antipyretic drugs were analyzed as well. Also, time-to-onset, time-to-recovery and perceived burden of AEFIs was compared between the sexes. Third, a literature review was done to retrieve sex-disaggregated outcomes of COVID-19 vaccination.

**Results:**

The cohort included 27,540 vaccinees (38.5% males). Females showed around two-fold higher odds of having any AEFI as compared to males with most pronounced differences after the first dose and for nausea and injection site inflammation. Age was inversely associated with AEFI incidence, whereas a prior COVID-19 infection, the use of antipyretic drugs and several comorbidities were positively associated. The perceived burden of AEFIs and time-to-recovery were slightly higher in females.

**Discussion:**

The results of this large cohort study correspond to existing evidence and contribute to the knowledge gain necessary to disentangle the magnitude of the effect sex in response to vaccination. Whilst females have a significant higher probability of experiencing an AEFI than males, we observed that the course and burden is only to a minor extent different between the sexes.

## Highlights

- Differences in response to vaccination between the sexes are generally well known. Yet, large studies specifically focusing on identifying differences between males and females with regard to reported adverse events and the course of adverse events are still scarce.
- In the Netherlands, the odds of experiencing an adverse event after COVID-19 vaccination is around two-fold higher in females compared to males for a range of different adverse events, while time-to-onset did not differ between the sexes and the time-to-recovery and perceived burden only slightly differed.
- Increasing knowledge on sexual dimorphism in response to vaccination can aid in providing more targeted safety information in future vaccination campaigns.

## 1 Introduction

The large scale vaccination of people around the world as preventive measure against COVID-19 infection has put more emphasis on safety and efficacy of vaccines. In the Netherlands, a vaccination campaign has been implemented since January 2021. Depending on age and the availability of vaccine brands during the vaccination schedule, people received one of the four vaccine brands authorized in Europe at that time, including the viral vector vaccines Oxford-AstraZeneca and Johnson&Johnson/Janssen, and the mRNA vaccines Moderna and BioNTech/Pfizer; hereafter referred to as AstraZeneca, Johnson&Johnson, Moderna and Pfizer respectively. Monitoring of vaccine safety (amongst others for the COVID-19 vaccines) allows for early detection of adverse reactions possibly linked to vaccination, thereby providing the first step in minimizing possible negative effects attributable to vaccines and allowing the provision of information about possible adverse reactions to vaccine recipients based on up-to-date data. This is achieved by The Netherlands Pharmacovigilance Centre Lareb through a spontaneous reporting system as well as longitudinal cohort event monitoring (CEM) studies (1, 2). AEFIs are defined as any unintended medical occurrence after immunization which is not necessarily causally related to the application of the vaccine (3). AEFIs that present as physical symptoms of the inflammatory response after vaccination, such as swelling, redness or pain at the injection site, headache, arthralgia or fever, are referred to as reactogenicity and constitute a significant part of all AEFIs reported (4). The types and degree of adverse events after immunization (AEFIs) differ between vaccine brands, with the Pfizer generally being associated with the lowest incidence of reported AEFIs (5–7). Apart from vaccine brand, several recipient-factors influence the probability of experiencing an AEFI. These include age and sex and also the presence of comorbidities such as diabetes, cardiovascular disorders and conditions/medications that suppress the function of the immune system (4, 8).

It is well recognized that most females mount stronger antibody responses against vaccination and experience a higher level of reactogenicity compared to males which is mainly attributable to levels of sex hormones and chromosomal differences (9, 10). In this context, ‘sex’ is referring to differences between individuals based on biological characteristics (i.e. sex organs, chromosomes, endogenous hormone profiles) rather than cultural and social aspects which more reflect a person’s self-representation and is often denoted as ‘gender’ (11). Sex is commonly controlled for in epidemiological studies rather than being the main topic of study. Nonetheless, the importance of reporting sex-disaggregated results of medical conditions and interventions is gaining attention, particularly since the COVID-19 pandemic where differences in responses to infection as well as safety and efficacy of vaccines could be studied in diverse and larger populations.

Several studies report a significant higher incidence of reported AEFIs after COVID-19 vaccination in females than males (12–14). Yet, only a limited number of studies focus specifically on the association between the occurrence of AEFIs and sex. Hence, much is still unknown about differences in the course of the AEFI and the sex-disaggregated effects of aging on AEFI occurrence. In this longitudinal cohort event monitoring study, we investigated the incidence of reported AEFIs between males and females after the first and second dose of vaccination against Covid-19 in a Dutch cohort covering four vaccine brands. Secondly, we aimed to assess whether the latency, duration and perceived burden of the reported AEFIs differed between males and females. Third, we reviewed existing literature assessing the incidences of AEFIs in males and females to allow for comparison with the results from our cohort study.

## 2 Methods

### 2.1 Data collection and cohort description

The design of the study and the process of data collection have been described in detail elsewhere (2, 5). Briefly, Dutch residents aged above 16 years who received the first dose of COVID-19 vaccination between February and August 2021 were eligible to participate in this prospective cohort study. Data were collected through web-based questionnaires using the Lareb Intensive Monitoring (LIM) system. At registration (at a maximum of two days after the first dose of vaccination), vaccinees filled in a baseline questionnaire covering questions about age, sex, body weight and length, presence of comorbidities, concomitant use of medication, the use of antipyretic drugs shortly before vaccination and whether the person had a history of COVID-19 infection before vaccination. Following the baseline questionnaire, participants received six questionnaires over a period of six months in which they could self-report the occurrence of AEFIs possibly attributable to their first or second COVID-19 vaccination. Moreover, the course (burden and time to recovery) of reported AEFIs was retrieved in the questionnaires. People who only completed the baseline questionnaire and none of the six follow-up questionnaires were excluded from analyses as well as people who received a different vaccine brand for their second dose vaccination compared to the first dose.

### 2.2 Classification of AEFIs and predictor variables

The AEFIs reported in the study were coded using the Medical Dictionary for Regulatory Activities (MedDRA^®^) terminology 23.0 and 24.0 (15). The incidence and course of reported AEFIs was compared between males and females for multiple outcomes for the first and second dose of vaccination separately. These outcomes include any reported AEFI (hereafter referred to as ‘any AEFI’), local reactions at the injection site and the 10 most frequently reported AEFIs regardless of sex. The MedDRA preferred terms classified as local reactions are listed in **Supplementary Table S1**. For all outcomes, the maximal time between vaccination and onset of the AEFI for an AEFI to be included in the statistical analyses was 28 days (≤672 hours). Age and body mass index (BMI) were included in the analyses as categorical variables (age groups <40, 40-54, 55-74, ≥75 years; BMI <18.5, 18.5-24.9, 25.0-29.9, ≥30.0). Underlying medical conditions were categorized in 12 variables comprising the presence/absence of allergies, cardiovascular disorders, diabetes, hepatic diseases, hypertension, renal diseases, respiratory diseases, malignant tumors, neurological disorders, psychological disorders, a suppressed immune function or other comorbidities. In addition, the use of antipyretic drugs in the hours before or after the first dose of vaccination and whether or not an individual had a PCR-confirmed COVID-19 infection before vaccination were included in the analyses as well. Also, participants could report the time to onset (TTO) of an AEFI and the time to recovery (TTR) in date format and/or a number of seconds, minutes, hours, days or weeks. For TTO analysis, we considered a reported TTO in seconds, minutes or hours as more precise than a date of onset or a reported number of days or weeks after vaccination, hence, we restricted the TTO analysis to only those AEFIs which were reported in seconds, minutes or hours. With regard to TTR analysis, we did not restrict on time unit as recovery is presumably more gradual than onset. The perceived burden was retrieved in a question with five answer options ranging from ‘not at all’, ‘slightly’, ‘somewhat’, ‘moderately’ to ‘extremely’ burdensome.

### 2.3 Statistical analysis

First, we made an overview of the AEFIs which were exclusively reported by one of the sexes. The AEFIs exclusively reported by one of the sexes were included in further analyses as well. Second, we performed univariate logistic regression for the outcome ‘any AEFI’ and local reactions on the whole cohort by dose to assess which variables might predict the occurrence of AEFIs. Subsequently, least absolute shrinkage and selection operator (LASSO) regression was applied to select the combination of variables used in further multivariable logistic regression for the outcomes ‘any AEFI’ and local reactions for the first and second dose separately, using the R glmnet package (16). For the multivariable logistic regression of the 10 most frequently reported AEFIs, a smaller subset of covariates was used, as the number of reported outcomes in these groups were lower. For all outcomes (any AEFI, local reactions and the top 10 most frequent), because the distribution of the time to onset data as well as the time to recovery data did not meet the assumptions of normality, we performed Kruskal-Wallis tests for ‘any AEFI’, local AEFIs and the top 10 most reported AEFIs by sex and vaccine separately. Subsequently, as we tested many correlations, we applied a Bonferroni correction to account for multiple testing to protect from Type I errors. As the proportional odds assumption was violated for the perceived burden data (i.e. indicating that the distance between each of the answer options is presumably not equal), we assessed the association between sex and burden by means of a multivariable logistic regression model using sex as outcome, and age, vaccine brand, and burden level as covariates. All analyses were done with R version 4.1.3. P-values <0.05 were considered significant.

### 2.4 Literature review

In addition to the data analysis of the longitudinal cohort, we performed a literature review to summarize the main outcomes of studies that previously investigated the difference in incidence of reported AEFIs between males and females after COVID-19 vaccination with one of the vaccine brands covered by our cohort study (i.e. AstraZeneca, Johnson&Johnson, Moderna, Pfizer). We systematically searched the PubMed database for articles reporting sex-disaggregated outcomes of adverse effects in cross-sectional studies, cohort studies, clinical trials and case-control studies, published between 2019 and May 2022. The search was conducted by combining several groups of search terms listed in **Supplementary Table S2** related to vaccine brand, study type and adverse effects and including at least one keyword referring to sex or one of the sexes in the title and/or abstract. Additional exclusion criteria comprised articles focusing on one specific AEFI or a selective population such as people with a specific underlying comorbidity (e.g. rheumatoid arthritis), those exclusively focusing on pregnant or lactating women or articles reporting the outcomes of pre-clinical or non-clinical studies (**Supplementary Table S3**). After removal of duplicates, stepwise exclusion of irrelevant articles was achieved by screening title, abstract and full text. Any disagreements in the selection process were solved through discussion between the reviewers to reach consensus. Subsequently, year of publication, country/countries of study, vaccine brands used, size of the study population and main outcomes were extracted from each of the included articles.

## 3 Results

### 3.1 Cohort description

The initial cohort consisted of 31,033 vaccinees who filled in the baseline questionnaire of which 38.6% were males. After exclusion of people who did not complete any of the follow-up questionnaires about the occurrence of AEFIs, 27,540 vaccinees remained for inclusion in the analysis. **Table 1** shows the number of male and female vaccinees who completed at least one follow-up questionnaire after the first and the second dose, by vaccine brand. For Johnson & Johnson and Moderna, about two-third of the vaccinees in the cohort were females, whereas for AstraZeneca the proportion of females was higher and for Pfizer the proportion was lower compared to males (**Table 1**). The average age differed by age and vaccine brand (**Table 1**), with generally a lower age for those who received the Johnson & Johnson vaccine and a higher age for those who received the Pfizer vaccine. About half of the vaccinees reported presence of one or more comorbidities, mainly hypertension (16.4%), allergies (11.0%) and other comorbidities not classified into one of the other categories (11.7%).

**Table 1:** Number and median age of vaccinees in the cohort who completed ≥1 questionnaire after the first and second dose.

	Number of vaccinees (%)			Median age (IQR)	
	Males	Females	Total	Males	Females
Dose 1					
AstraZeneca	1272 (14.5)	7503 (85.5)	8775	58 (41-64)	48 (36-58)
Johnson&Johnson	698 (28.4)	1756 (71.6)	2454	48 (36-54)	45 (36-53)
Moderna	1155 (33.7)	2274 (66.3)	3429	49 (41-55)	45 (35-52)
Pfizer	7486 (58.1)	5396 (41.9)	12,882	78 (74-81)	71 (41-78)
**Total**	10,611 (38.5)	16,929 (61.5)	27,540	74 (53-79)	50 (37-62)
Dose 2					
AstraZeneca	836 (15.1)	4707 (84.9)	5543		
Johnson&Johnson					
Moderna	881 (33.9)	1718 (66.1)	2599		
Pfizer	6601 (60.4)	4333 (39.6)	10,934		
**Total**	8318 (43.6)	10,758 (56.4)	19,076		

IQR, interquartile range.

### 3.2 Frequencies of AEFIs reported by males and females

A total of 4774 males and 13857 females reported at least one AEFI within 4 weeks after the first dose of vaccination, which corresponds to 45.0% and 81.9% of the male and female vaccinees respectively. The higher proportion of females compared to males who reported an AEFI was consistent for both the first and second dose for all four vaccine brands. Three sex-restricted AEFIs (at MedDRA lower level term) were reported by males, including erectile failure, pain in testis and scrotum swelling, whereas in females a variety of sex-restricted AEFIs were reported, mostly associated with the menstrual cycle or menstruation (**Supplementary Table S4**).

### 3.3 Factors influencing the probability of experiencing an AEFI

In the univariate regression analysis, most of the variables included in the analysis (n=17/19) appeared significantly associated with the occurrence of ‘any AEFI’ (**Supplementary Table S5**). In the univariate analyses, the odds of having ‘any AEFI’ was 5.52 times higher in females compared to males (95%CI 5.22-5.83; p<0.001; **Supplementary Table S5**) after the first dose, whereas the OR for having a local AEFI was 3.18 (95%CI 3.02-3.36; p<0.001; **Supplementary Table S6**). Both ORs for any AEFI and local reactions were less pronounced after the second dose (any AEFI 2.74; 95%CI 2.58-2.91; local reaction 2.35; 95%CI 2.19-2.52; **Supplementary Tables S5**, **S6**). The effect of age and vaccine brand was similar in both sexes. The probability of experiencing an AEFI decreased by age in both males and females (**Supplementary Figure S1**). Furthermore, the odds of having an AEFI was higher after COVID-19 infection, yet, this association was stronger for males compared to females (ORs first dose 3.46 [95%CI 2.76-4.37] *vs.* 2.59 [95%CI 2.11-3.21]; ORs second dose 1.93 [95%CI 1.50-2.48] *vs.* 1.19 [95%CI 1.02-1.39]). Similarly, the use of antipyretic drugs shortly before vaccination was associated with a higher odds of having an AEFI in males than in females (ORs 2.85 [95%CI 2.42-3.38] *vs.* 1.99 [95%CI 1.77-2.25]).

Lasso regression on ‘any AEFI’ as outcome showed that sex, age group, confirmed COVID-19 infection in the past, the use of antipyretic drugs, and seven comorbidities were eligible variables for inclusion in the multivariable model for the first dose. For the data of the second dose, the same variables appeared eligible for the multivariable model with the exception of confirmed COVID-19 infection in the past, the use of antipyretic drugs, hypertension and a suppressed immune function. In these multivariable models, the odds of having any AEFI in females compared to males was 2.28 (95%CI 2.13-2.43; p<0.001) after the first dose and 2.02 (95%CI 1.87-2.17; p<0.001) after the second dose. While far less vaccinees reported an AEFI after the second dose of vaccination with AstraZeneca or Pfizer compared to the first dose, no difference was observed in the occurrence of an AEFI between the first and second dose of Moderna vaccination for both males and females (**Table 2**). Six out of the seven comorbidity variables were associated with a significant higher odds of having an AEFI. For the local reactions, the effects of sex, age, prior confirmed COVID-19 infection and comorbidities were somewhat less pronounced compared to ‘any AEFI’ for the for the first dose. Whilst the odds of experiencing ‘any AEFI’ in people who received the first dose of Moderna *versus* the AstraZeneca vaccine was 0.37, the odds of having a local reaction was higher for those who received the Moderna *versus* AstraZeneca vaccine (OR 1.22).

**Table 2:** Multivariable logistic regression of factors associated with the occurrence of ‘any AEFI’ and local reactions, by dose.

	Any AEFI		Local reactions	
	Dose 1	Dose 2	Dose 1	Dose 2
	aOR^a^ (95%CI)	aOR^b^ (95%CI)	aOR^c^ (95%CI)	aOR^d^ (95%CI)
Sex				
Males	–	–		
Females	2.28 (2.13-2.43)***	2.02 (1.87-2.17)***	1.91 (1.79-2.02)***	1.82 (1.68-1.97)***
Age				
<40 years	–		–	–
40-54 years	0.39 (0.35-0.44)***	0.51 (0.46-0.57)***	0.92 (0.86-0.99)*	0.82 (0.74-0.90)***
55-74 years	0.19 (0.17-0.22)***	0.29 (0.26-0.32)***	0.60 (0.55-0.64)***	0.56 (0.51-0.62)***
≥75 years	0.10 (0.09-0.11)***	0.17 (0.15-0.19)***	0.30 (0.27-0.33)***	0.33 (0.29-0.37)***
Vaccine				
AstraZeneca	–			
Johnson&Johnson	0.32 (0.28-0.37)***	–	0.48 (0.43-0.52)***	
Moderna	0.37 (0.32-0.41)***	6.81 (5.99-7.75)***	1.22 (1.12-1.32)***	3.69 (3.33-4.09)***
Pfizer	0.19 (0.17-0.21)***	1.52 (1.39-1.66)***	0.64 (0.59-0.69)***	1.49 (1.5-1.64)***
**Conf. COVID-19 infection**	2.11 (1.78-2.51)***	–	1.49 (1.34-1.67)***	–
**Use of antipyretic drug**	1.35 (1.20-1.50)***	–	–	–
**Allergy**	1.75 (1.56-1.96)***	1.50 (1.35-1.68)***	1.33 (1.22-1.44)***	1.28 (1.15-1.42)***
**Cardiovascular disorders**	1.26 (1.14-1.39)***	1.18 (1.07-1.31)***	–	
**Hypertension**	0.92 (0.85-0.996)*	–	–	
**Psychological disorders**	1.59 (1.29-1.97)***	1.32 (1.09-1.60)**	1.31 (1.14-1.49)***	1.37 (1.14-1.63)***
**Respiratory diseases**	1.14 (1.02-1.28)*	1.19 (1.06-1.33)**	–	
**Suppressed immune function**	1.38 (1.09-1.74)**	–	–	
**Other comorbidities**	1.42 (1.28-1.56)***	1.29 (1.17-1.42)***	1.27 (1.17-1.38)***	1.40 (1.27-1.55)***

aOR, adjusted odds ratio. Conf.: PCR-confirmed. *p<0.05, **p<0.01, ***p<0.001.

^a^ Adjusted for: sex, age group, vaccine, confirmed prior COVID-19 infection, preventive use of antipyretic drugs, allergy, cardiovascular disorder, hypertension, respiratory diseases, psychological disorders, suppressed immune function and other comorbidities.

^b^Adjusted for: sex, age group, vaccine, allergy, cardiovascular disorder, respiratory disease, psychological disorders and other comorbidities.

^c^Adjusted for: sex, age group, vaccine, confirmed prior COVID-19 infection, allergy, psychological disorders and other comorbidities.

^d^Adjusted for: sex, age group, vaccine, allergy, psychological disorders and other comorbidities.

**Figure 1:**
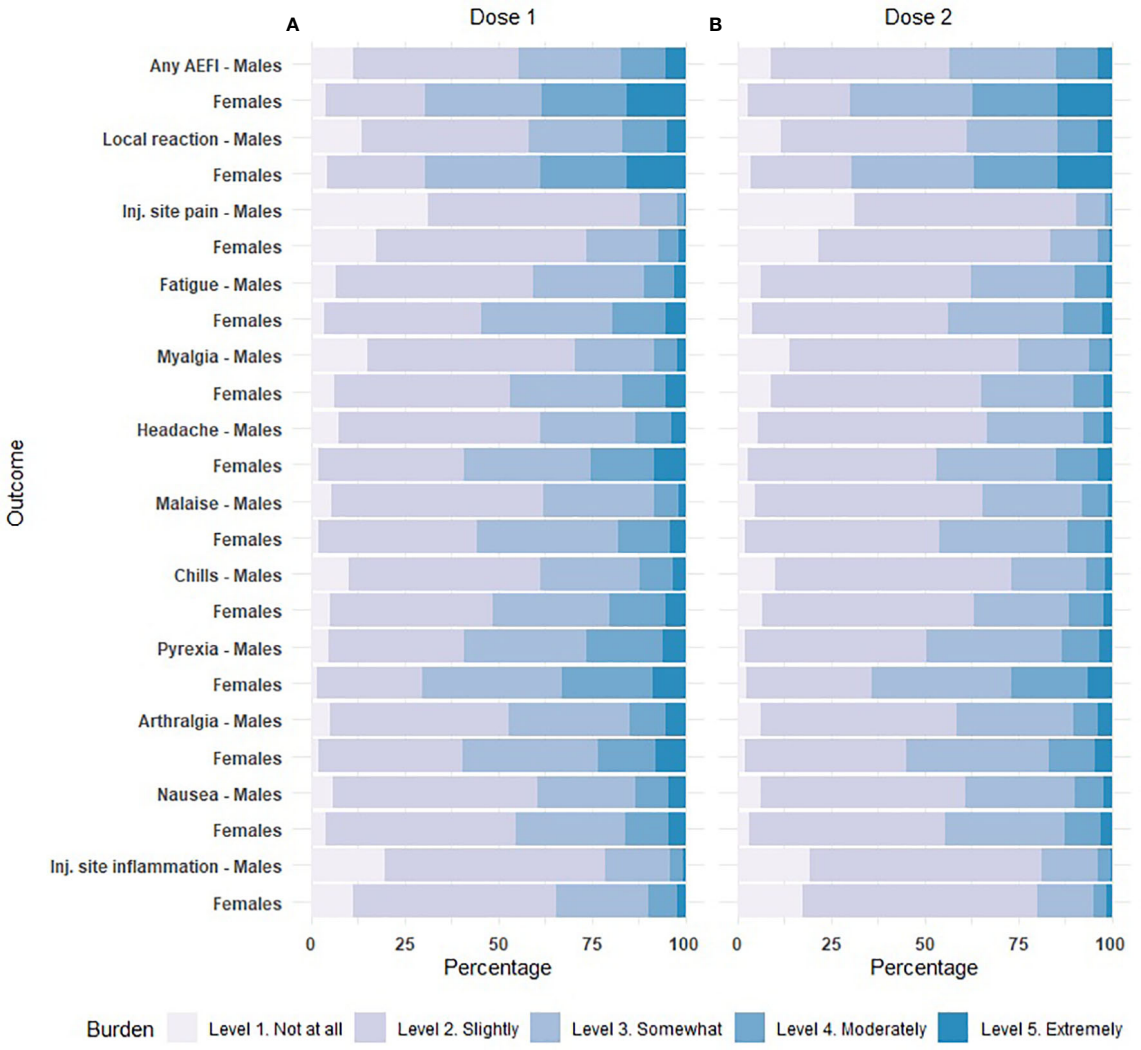
Forest plot of the odds of experiencing an adverse event after immunization (AEFI) in females versus males after dose 1 and dose 2, based on multivariable logistic regression. The size of the square corresponds to the inverse of the standard error. aOR: adjusted odds ratio. Inj.: injection. * p<0.05, **p<0.01, ***p<0.001. **(A)**. Adjusted for: sex, age group, confirmed prior COVID-19 infection, preventive use of antipyretic drugs. **(B)**. Adjusted for: sex, age group.

The 10 most frequent reported AEFIs (regardless of sex) consist of two local reactions and eight systemic reactions and all were solicited adverse effects. **Figure 1** shows the ORs for the occurrence of the 10 AEFIs in females versus males. Overall, the odds of developing any of the 10 AEFIs was around two-fold higher in females compared to males. The ORs were similar for the first and second dose, with the largest difference between the first and second dose for nausea and injection site inflammation. For both doses of vaccination, the least difference in odds between males and females was found for myalgia, with ORs ranging between the different vaccine brands of 1.16-1.56 and 1.52-1.65 for the first and second dose respectively. The highest differences between males and females were observed for nausea (OR range 2.39-4.05) and injection site inflammation (OR range 2.29-4.00). The difference in odds between males and females was lowest for the Moderna vaccine and highest for the Pfizer vaccine for most AEFIs. Particularly for nausea and chills, the ORs for females *versus* males who received the Pfizer vaccine deviated substantially from the other vaccine brands (**Figure 1**).

### 3.4 Course of reported AEFIs

#### 3.4.1 Time to onset and time to recovery

We compared the TTO of ‘any AEFI’, local reactions and the top 10 most reported AEFIs between males and females only for those AEFIs which included a TTO in seconds, minutes or hours (as this was considered more precise than only a date of onset). Overall, for 27.3% of the AEFIs (reported ≤28 days after vaccination) such detailed latency information was provided, which were slightly more originating from females vaccinees compared to males (28.0% *vs.* 24.3) and for the first dose as compared to the second dose (29.6% *vs.* 21.2%). Also, the proportion of AEFIs with detailed latency information was lower for the Moderna (23.1%) and Pfizer vaccines (23.6%) as compared to Johnson&Johnson (32.0%) and AstraZeneca (30.2%). For over 95% of the reported AEFIs the time to recovery (TTR) was reported. **Supplementary Table S7** displays the median and interquartile range (IQR) of the reported TTO and TTR of the AEFIs by sex, dose and vaccine brand. Overall, little difference existed between the TTO of the AEFIs reported by males and females. For both sexes, shorter TTOs were observed for local reactions (medians of 3 and 2 hours for males and females respectively) compared to the systemic reactions such as pyrexia, nausea and chills were the median latency times frequently exceeded eight hours. In contrast to TTO, the TTR lasted significantly longer in females than males for several AEFIs (**Supplementary Table S7**). Females suffered significantly longer from ‘any AEFI’, local reactions, injection site pain and inflammation, fatigue, headache and malaise compared to males when using data from all vaccine brands combined. For most AEFIs the TTR lasted 1-3 days, the longest TTR was reported for injection site inflammation with medians of 72 and 96 hours in males and females respectively. For most AEFIs the median TTRs were equal or lower after the second dose compared the first dose, while no such tendency was observed for TTO.

#### 3.4.2 Perceived burden

**Figure 2:**
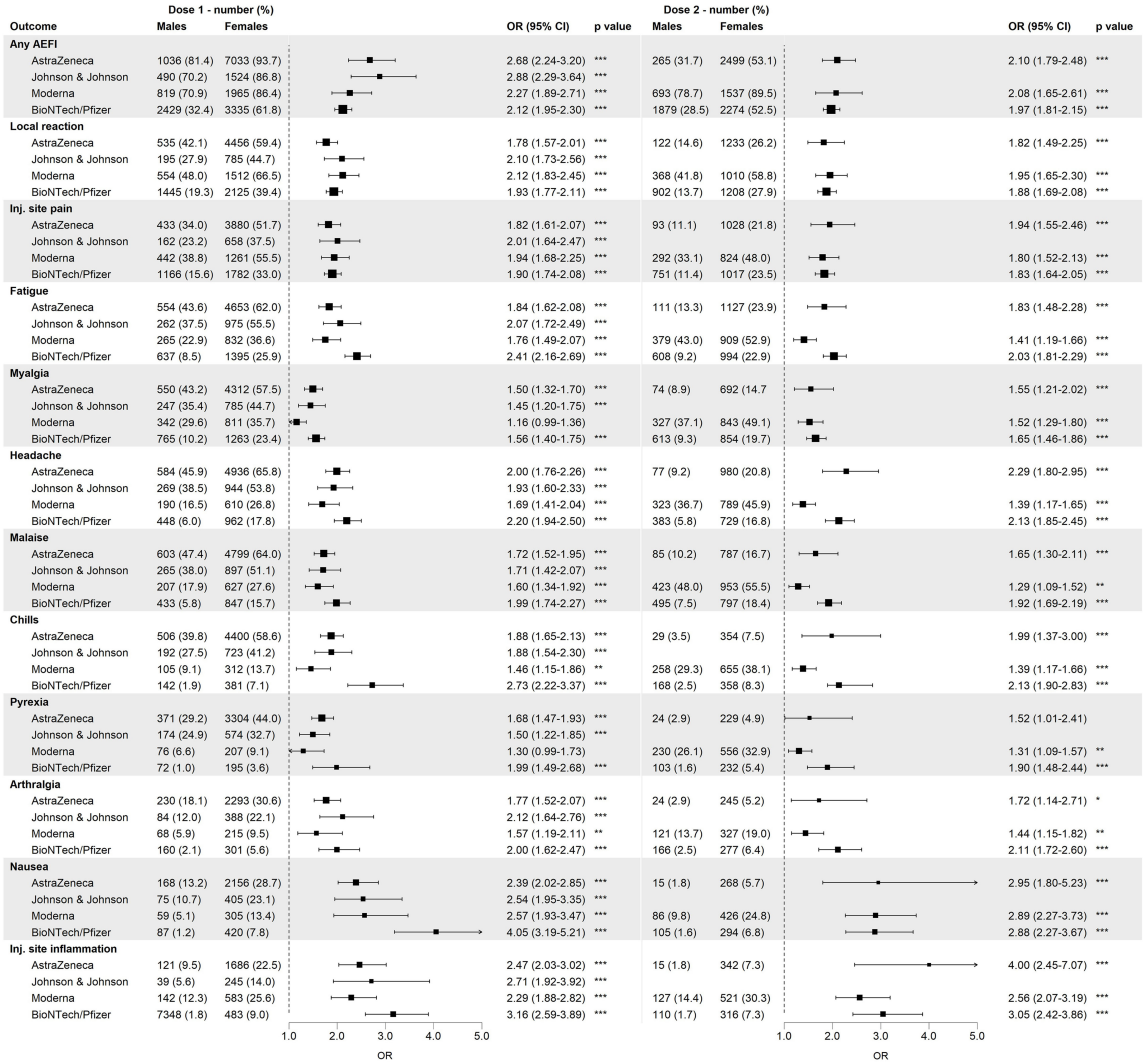
Distribution of the burden classes among males and females among different adverse events following immunization (AEFIs), based on data of the first dose and all vaccine brands combined. Inj.: injection.

For over 97% of the reported AEFIs, the perceived burden was reported. **Figure 2** shows the proportions for all five burden levels for any AEFI, local reactions and the 10 most reported AEFIs by sex, based on the maximal burden level reported by each person for the respective outcome. For all outcomes, a higher fraction of males compared to females reported the lowest burden level (‘Not at all burdensome’), whereas the opposite was true for the highest burden levels. Injection site pain was considered not very burdensome by both males and females.

The multivariable logistic regression showed that for ‘any AEFI’, vaccinees who reported a burden level of 2, 3, 4 and 5 had respectively 1.40, 2.13, 2.92 and 4.39 times higher odds of being female as compared to vaccinees who reported the lowest burden level (‘Not at all burdensome’) (**Supplementary Figure S2**), indicating that with each step increase in burden level, the fraction of females compared to males increased as well. This trend was observed for most AEFIs though with a varying magnitude of the association between sex and burden for the studied AEFIs as well as the two doses. The reported burden levels of nausea were not significantly associated with sex, neither was the burden of pyrexia and injection site inflammation after the second dose of vaccination. In contrast, ORs for arthralgia after the second dose were high as a relative small portion of females reported the arthralgia being not at all burdensome as compared to males.

### 3.5 Literature review

The literature search resulted in 436 unique potentially relevant articles. After exclusion of 340 irrelevant articles based on title or abstract, 96 articles were subjected to full-text screening of which 84 were eligible to be included in the review (**Supplementary Figure S3** and **Table S8).** Forty-three of these articles concerned cross-sectional studies (51.2%) (12, 13, 17–57), 26 were prospective cohort studies (31.0%) (2, 5, 14, 58–80), eight retrospective cohort studies (9.5%) (81–88) and seven were other types of studies (6, 89–94) (8.3%). The majority of the studies were performed in Europe (n=26, 31.0%), in Asia (n=24, 28.6%) and in the Middle East (n=21, 25.0%). With regard to the vaccine brands investigated, the highest percentage of the articles under review assessed the occurrence of AEFIs after Pfizer vaccination (n=71, 84.5%), followed by AstraZeneca (n=34, 40.5%), Moderna (n=24, 28.6%), and Johnson&Johnson (n=9, 10.7%) vaccines (**Supplementary Table S8**).

Nearly all articles reported a higher incidence of AEFIs in females compared to males with the exception of four articles which found an opposite outcome, for most of which no clear reason could be identified other than a possible skewed distribution of the sexes and age groups in the studies (17, 18, 80, 85). Of the 21 articles which reported a OR of any adverse reaction for sex, the median OR was 1.93 (range: 0.85-3.45, IQR 1.49-2.50) using males as reference group (**Supplementary Table S8**) (2, 6, 13, 22, 24, 25, 34, 43, 45–47, 52, 54, 56, 61, 63, 66, 68, 79, 81, 85). Similarly, females had a median 1.96 times higher odds of reporting a local reaction compared to males (range: 1.02-2.90, IQR1.85-2.54) (22, 32, 42, 54, 76). With regard to specific common AEFIs, median ORs of 2.42 (range 2.07-4.72), 2.01 (range 1.57-2.61), and 1.77 (range 1.63-1.84) were reported for headache (32, 51, 67, 76), fatigue (22, 32, 51, 67, 76), and fever (32, 68, 76) respectively. TTR was assessed in four articles, all of which reported higher TTR in females compared to males ranging from 1.2-1.9 days in males to 1.4- 2.2 days in females, though the difference was only statistically significant in one article (23, 31, 54, 66). The results in the articles regarding health care seeking behaviour, hosptial admissions and absenteeism due to adverse effects were unambiguous across studies. Females showed a higher rate of health care seeking behavior and absenteeism in two studies (30, 86), whereas two studies reported higher hospital admission rates for males (87, 92).

## 4 Discussion

In this nationwide prospective cohort study, using patient reported outcomes, we assessed the incidences of reported AEFIs after COVID-19 vaccination in males and females and compared the reported TTO, TTR and perceived burden between the sexes. Also, we summarized the literature which reported sex-disaggregated outcomes concerning possible adverse reactions after vaccination with one of the vaccine brands included in our cohort study.

In the past, women were underrepresented in clinical trials and sex-disaggregated results were often not published (95). Current clinical study guidelines recommend that trials include an adequate demographic characterization of the patient/target population, including a representative sex distribution, and that analyses of safety and efficacy data will be stratified on sex, as one cannot assume the absence of sexual dimorphism in the effects of vaccination/drugs (96, 97). Large clinical trials for COVID-19 vaccines have accordingly included a proportionate number of females, however, a literature review showed that 30% of the studies with efficacy data and 34% of the studies with safety data presented sex-disaggregated outcomes (98, 99).

Consistent with the existing body of literature which mainly included post-marketing studies, females showed an around two-fold higher incidence of AEFIs than males in our cohort. The difference was most pronounced after the first dose and for the AEFIs nausea and injection site inflammation. Several factors might contribute to this dimorphism in AEFI incidence including biological differences between both sexes. Compared to males, females show higher humoral and cell-mediated immune responses to vaccination. Binding of sex hormones such as estrogens to specific receptors on immune cells, including dendritic cells, macrophages and lymphocytes, affects signaling pathways involved in the production of chemokines and cytokines ultimately inducing a stronger immune response (9). In contrast, the activity of immune cells is suppressed by testosterone and dihydrotestosterone (100). Beyond sex hormones, the X chromosome expresses 10-fold more genes related to immunity compared to the Y chromosome and polymorphisms in genes on the sex chromosomes can affect immune responses, which explains the presence of differences between the sexes before the reproductive age and after reproductive senescence (9).

Moreover, for some reported AEFIs comprising physical complaints not restricted to vaccination such as headache and nausea, a substantial difference in background incidence (i.e. regardless of vaccination status) exists between both sexes which diluted or fortified the observed effect of vaccination. For instance, over two-fold higher background incidences of headache have been documented in females (101, 102). Similarly, females tend to suffer more from nausea than males in other contexts such as motion sickness or after general anesthesia which is hypothesized to be associated with sexual dimorphisms in availability of neurokinin-1 receptors involved in vomiting and nausea (103).

In both males and females a clear inverse association between age and the probability of experiencing AEFIs was observed owing to the process of immunosenescence (104). Although the effect of aging is stronger, at a higher pace and with an earlier onset in males than females (105, 106), this was not reflected by the incidence of AEFIs in our cohort. The outcomes of multivariable logistic regression showed significant positive associations between occurrence of ‘any AEFI’ and presence of several comorbidities, with ORs varying between 1.14 and 1.75. As for part of these associations a direct causal relationship is lacking, this could be subject of future studies to further elucidate these findings. The use of antipyretic drugs shortly before or after vaccination was associated with a higher odds of having ‘any AEFI’. Although the preventive use of antipyretic drugs would logically assume a lower incidence of AEFIs, the positive association can be the result of the phrasing of the question as people could have taken antipyretic drugs to suppress the symptoms of early AEFI(s) occurring within a few hours after vaccination.

The median TTO and TTR were in line with existing literature although results were not very consistent across studies (23, 24, 31, 54, 66, 83). For the articles which mentioned the TTR, the differences between males and females were generally less than one day (31, 54, 66, 83). The perceived burden of the AEFIs was significantly different between males and females, yet, comparing our results with other studies is difficult as only few studies incorporated a burden or severity indicator and mostly these are based on absenteeism or hospital admission rather than on a perceived scale.

We separately reported on sex-specific AEFIs. Over 300 females reported one or multiple menstrual disorders after the first and/or second dose of vaccination and an additional 13 females reported postmenopausal hemorrhage. The Netherlands Pharmacovigilance Centre Lareb has previously reported on data on menstrual disorders and post-menopausal bleeding, both from the spontaneous reporting system and this cohort (107, 108).

The strengths of this study includes the large study population size and availability of information on immunization status, vaccine brand, comorbidities and COVID-19 infection history. By using patient reported outcomes we ensure that also AEFIs for which no medical attention is needed are captured. Yet, for the interpretation of the outcomes of this study, some limitations should be taken in consideration. We confined the period of an AEFI to be attributed to vaccination to 28 days as most AEFIs occur within the first days to weeks after vaccination. Choosing a narrower time interval could have resulted in AEFIs potentially being missed, whereas a broader time interval would have interfered with the second dose of vaccination. Nonetheless, we might have missed AEFIs with a longer latency time and might have attributed AEFIs with a long latency time incorrectly to the second vaccine dose in case the onset of the AEFI was after the date of second vaccination. Another limitation is the fact that we likely missed several articles in the literature review which reported sex-disaggregated results in the main body of the article but not in the abstract (to which we restricted our search), although this probably does not affect the overall summarized outcomes. Noteworthy, in our analysis, the AEFI pyrexia (at MedDRA PT level) included people with a maximum body temperature of 38-40.5°C or those with fever without measurements. In other studies, the coding or questionnaires might have been different.

In conclusion, this study presents sex-disaggregated outcomes of adverse effects following COVID-19 vaccination of four different vaccine brands. The incidence of any AEFI, local reactions as well as the top 10 most frequent AEFIs was higher in females compared to males for both doses. Also, the results of this cohort study showed that the TTR and the perceived burden differed to a minor extent between both sexes. Our results confirm the outcomes of previous studies and aid in the gain of knowledge with regard to differences in response to vaccination between males and females.

## Data availability statement

All data relevant to the study are included in the article or uploaded as **Supplementary Information**.

## Ethics statement

Dutch regulations oblige all studies in the Netherlands subjected to the Medical Research Involving Human Subjects Act (WMO) to be reviewed by an accredited Medical Ethics Committee (METC) or the central committee on research involving human subjects (CCMO). The board of the METC Brabant reviewed this study and concluded that the rules included in the WMO act did not apply to this study, hence, this study was considered exempt from the requirement for approval.

## Author contributions

AK, FH, JD, TL, LB, MR, and TL designed the study part covering analysis of cohort data. TL and LB: performed data cleaning. TL and JD performed data preparation and pre-analysis data coding. JD performed statistical data analysis. FH, JD, and SP designed the literature review. SP performed the literature search. JD, FH, and SP original draft preparation. All authors contributed to the article and approved the submitted version.
