# Supplementary material for: Sex-disaggregated outcomes of adverse events after COVID-19 vaccination: A Dutch cohort study and review of the literature
Source: Front Immunol. 2023 Jan 30;14:1078736. doi: 10.3389/fimmu.2023.1078736 (PMC9922710; doi:10.3389/fimmu.2023.1078736)
Supplement: Supplementary file 1 [file DataSheet_1.docx]

# **Supplementary tables and figures**

**Table S1**. Preferred terms classified as local reactions.

| **MedDRA preferred term** | |
| --- | --- |
| - Extensive swelling of vaccinated limb - Injection site abscess - Injection site bruising - Injection site cellulitis - Injection site discoloration - Injection site discomfort - Injection site dryness - Injection site eczema - Injection site erythema - Injection site exfoliation - Injection site extravasation - Injection site haematoma - Injection site haemorrhage - Injection site hypoaesthesia - Injection site induration - Injection site infection - Injection site inflammation - Injection site irritation | - Injection site joint movement impairment - Injection site joint pain - Injection site mass - Injection site oedema - Injection site pain - Injection site papule - Injection site paraesthesia - Injection site pruritus - Injection site rash - Injection site reaction - Injection site swelling - Injection site urticaria - Injection site vesicles - Injection site warmth - Vaccination site dryness - Vaccination site lymphadenopathy - Vaccination site mass - Vaccination site paraesthesia |

**Table S2**. Search terms used in the literature review on sex-disaggregated outcomes of reported AEFIs after COVID-19 vaccination

| **Category 1 †** [Title/Abstract] | **Category 2 †**  [Title] | **Category 3 †**  [Title/Abstract] | **Category 4 †**  [Title/Abstract] | **Category 5†**  [Title/Abstract] | **Category 6 †**  [Title] |
| --- | --- | --- | --- | --- | --- |
| - sex - gender - men - females - males - females - sexual | - coronavirus - SARS-CoV-2 - SARS-CoV2 - Covid-19 - Covid19 - pandemic* | - AstraZeneca - Vaxzevria - ChAdOx1 nCoV-19 - AZD1222 - Janssen - Johnson & Johnson - Ad26.COV2.S - Pfizer - Pfizer’s Comirnaty - Pfizer-BioNTech - Pfizer/BioNTech - BNT162b2 - mRNA vaccin* - Moderna - Moderna’s SpikeVax - mRNA-1273 | - undesirable effect* - adverse effect* - adverse reaction* - side effect* - ADR - ADRs - adverse event* - AE - AEs - AEFI AEFIs - AESI* - important medical event - reactogenicit* - injection site reaction* - local reaction* | - immunisation - immunization - vaccin* | - review - meta-analysis - pre-clinical - non-clinical - non-human - animal - hesitancy - hesitation - acceptance - pregnant females - breastfeeding females - lactating females - man - male - woman - female - case report* |

**†** Key words in each category combined with **‘OR’**, categories 1-5 combined with **‘AND’** and category 6 combined with **‘AND NOT’**. Asterisks (*) indicates the use of truncation.

**Table S3.** Inclusion and exclusion criteria for the literature review on sex-disaggregated outcomes of reported AEFIs after COVID-19 vaccination.

| **Inclusion criteria** | **Exclusion criteria** |
| --- | --- |
| - One of the following study designs: - clinical trial - randomized controlled trial - case/clinical series - cohort studies - cross-sectional studies - case-control studies - Reporting one or more AEFIs/AESI/serious adverse events - Reporting sex-disaggregated outcomes of experienced AEFIs. | - Meta-analyses - Scoping reviews - Literature reviews - Systematic reviews - Pharmaceutical quality studies - Non-clinical/pre-clinical studies - Articles without sex-/gender-disaggregated data in outcomes - Case report(s) - Articles about hesitancy/acceptance or uptake rather than the actual experienced AEFIs - Articles focusing on pregnant/breastfeeding/lactating females - Articles focusing on one specific AEFI - Articles with a selective study population with underlying disease. |

**Table S4.** Number of sex-specific AEFIs reported after first and second dose combined^*^, by vaccine brand

|  | **AstraZeneca** | **Johnson& Johnson** | **Moderna** | **Pfizer** | **Total** |
| --- | --- | --- | --- | --- | --- |
| **FEMALES** |  |  |  |  |  |
| Absence of menstruation (amenorrhea) | 8 | 5 | 12 | 13 | 38 |
| Irregular menstrual cycle | 8 | 3 | 8 | 8 | 27 |
| Prolonged menstrual cycle | 1 |  | 2 | 3 | 6 |
| Shortened menstrual cycle | 7 | 7 | 18 | 25 | 57 |
| Menstrual interval <21 days (polymenorrhea) |  | 1 |  |  | 1 |
| Delayed menstruation | 8 | 7 | 16 | 24 | 55 |
| Abnormal menstruation | 1 | 1 | 1 | 1 | 4 |
| Prolonged menstruation | 14 |  | 9 | 12 | 35 |
| Shortened menstruation | 2 |  |  | 1 | 3 |
| Increased menstruation |  | 1 |  |  | 1 |
| Heavy menstrual bleeding (menorrhagia) | 18 | 11 | 13 | 24 | 66 |
| Low menstrual bleeding (hypomenorrhea) | 6 | 1 | 1 | 4 | 12 |
| Intermenstrual bleeding | 18 | 4 | 10 | 16 | 48 |
| Menstrual cramps (dysmenorrhea) | 1 | 3 | 2 | 11 | 17 |
| Menstrual discomfort | 1 |  |  | 1 | 2 |
| Menstrual disorder NOS |  | 1 |  |  | 1 |
| Premenstrual pain | 1 |  | 1 | 1 | 3 |
| Premenstrual breast pain |  | 1 |  |  | 1 |
| Premenstrual syndrome |  |  |  | 2 | 2 |
| Ovulation pain |  |  | 1 |  | 1 |
| Abnormal vaginal discharge | 3 |  |  | 2 | 5 |
| Vaginal candidiasis | 2 |  | 1 | 1 | 4 |
| Vulvovaginal pruritus |  |  | 1 |  | 1 |
| Vulval rash |  |  |  | 1 | 1 |
| Imbalance in vaginal flora | 1 |  |  |  | 1 |
| Menopausal symptoms aggravated | 1 |  |  |  | 1 |
| Menopause delayed | 1 |  |  |  | 1 |
| Postmenopausal bleeding | 4 | 3 | 3 | 3 | 13 |
| Breast pain |  |  | 2 | 2 | 4 |
| Mastitis | 1 |  |  |  | 1 |
| Lactation decreased | 1 |  |  |  | 1 |
| Lactation disorder |  |  |  | 3 | 3 |
| Missed abortion | 1 |  |  |  | 1 |
| Pregnant |  |  | 1 |  | 1 |
| Total females | 109 | 49 | 102 | 158 | 418 |
| **MALES** |  |  |  |  |  |
| Erectile failure |  |  |  | 1 |  |
| Pain in testis |  | 1 |  |  |  |
| Scrotum swelling |  |  |  | 1 |  |
| Total males |  | 1 |  | 2 | 3 |

AEFI: adverse event after immunization. NOS: not otherwise specified. *Without latency time restriction.

**Table S5.** Univariate logistic regression of factors potentially associated with the occurrence of ‘any AEFI’

|  | **Dose 1** |  |  | **Dose 2** |  |  |
| --- | --- | --- | --- | --- | --- | --- |
| Any AEFI | Reported ≥1 AEFI (%) | Crude OR (95%CI) | p-value | Reported ≥1 AEFI (%) | Crude OR (95%CI) | p-value |
| **Sex** |  |  |  |  |  |  |
| Males | 4774 (45.0) |  |  | 2837 (34.1) |  |  |
| Females | 13857 (81.9) | 5.52 (5.22-5.83) | <0.001 | 6310 (58.7) | 2.74 (2.58-2.91) | <0.001 |
| **Age** |  |  |  |  |  |  |
| <40 years | 5805 (92.1) |  |  | 2495 (75.8) |  |  |
| 40-54 years | 5846 (84.5) | 0.47 (0.42-0.52) | <0.001 | 2433 (65.6) | 0.61 (0.55-0.68) | <0.001 |
| 55-74 years | 4850 (68.7) | 0.19 (0.17-0.21) | <0.001 | 2437 (43.8) | 0.25 (0.23-0.27) | <0.001 |
| ≥75 years | 2130 (29.3) | 0.04 (0.03-0.04) | <0.001 | 1782 (27.4) | 0.12 (0.11-0.13) | <0.001 |
| **Vaccine** |  |  |  |  |  |  |
| AstraZeneca | 8069 (92.0) |  |  | 2764 (49.9) |  |  |
| Johnson&Johnson | 2014 (82.1) | 0.40 (0.35-0.46) | <0.001 | - | - | - |
| Moderna | 2784 (81.2) | 0.38 (0.34-0.42) | <0.001 | 2230 (85.8) | 6.08 (5.38-6.87) | <0.001 |
| Pfizer | 5764 (44.7) | 0.07 (0.07-0.08) | <0.001 | 4153 (38.0) | 0.62 (0.58-0.66) | <0.001 |
| **BMI** |  |  |  |  |  |  |
| <18.5 | 331 (80.7) |  |  | 170 (64.6) |  |  |
| 18.5-24.9 | 9544 (69.6) | 0.55 (0.42-0.70) | <0.001 | 4792 (51.0) | 0.57 (0.44-0.73) | <0.001 |
| 25.0-29.9 | 5753 (62.5) | 0.40 (0.31-0.51) | <0.001 | 2825 (43.0) | 0.41 (0.32-0.53) | <0.001 |
| ≥30.0 | 2802 (71.5) | 0.60 (0.46-0.77) | <0.001 | 1272 (47.9) | 0.50 (0.38-0.65) | <0.001 |
| Unknown | 201 (69.3) | 0.54 (0.38-0.76) | <0.001 | 88 (47.6) | 0.50 (0.34-0.73) | <0.001 |
| **Conf. COVID-19 infection** | |  |  |  |  |  |
| No | 17261 (66.5) |  |  | 8573 (47.4) |  |  |
| Yes | 1370 (87.2) | 3.42 (2.95-3.99) | <0.001 | 574 (59.1) | 1.60 (1.41-1.83) | <0.001 |
| **Use of antipyretic drug** | |  |  |  |  |  |
| No | 15396 (64.8) |  |  | - | - | - |
| Yes | 3224 (85.3) | 3.15 (2.87-3.47) | <0.001 | - | - | - |
| **Any comorbidity** | |  |  |  |  |  |
| No | 10302 (72.2) |  |  | 4655 (50.8) |  |  |
| Yes | 8329 (62.7) | 0.65 (0.62-0.68) | <0.001 | 4492 (45.3) | 0.80 (0.76-0.85) | <0.001 |
| **Allergy** |  |  |  |  |  |  |
| No | 16101 (65.7) |  |  | 7868 (46.0) |  |  |
| Yes | 2530 (83.5) | 2.64 (2.39-2.92) | <0.001 | 1279 (64.6) | 2.14 (1.95-2.36) | <0.001 |
| **Cardiovascular disorder** | |  |  |  |  |  |
| No | 17458 (70.2) |  |  | 8358 (49.8) |  |  |
| Yes | 1173 (44.1) | 0.33 (0.31-0.36) | <0.001 | 789 (34.6) | 0.54 (0.49-0.59) | <0.001 |
| **Diabetes** |  |  |  |  |  |  |
| No | 18052 (68.8) |  |  | 8797 (48.9) |  |  |
| Yes | 579 (45.0) | 0.37 (0.33-0.42) | <0.001 | 350 (32.8) | 0.51 (0.45-0.58) | <0.001 |
| **Hepatic disease** | |  |  |  |  |  |
| No | 18583 (67.6) |  |  | 9120 (47.9) |  |  |
| Yes | 48 (69.6) | 1.09 (0.66-1.86) | 0.734 | 27 (56.2) | 1.40 (0.79-2.50) | 0.251 |
| **Hypertension** | |  |  |  |  |  |
| No | 16462 (71.5) |  |  | 7831 (51.0) |  |  |
| Yes | 2169 (48.0) | 0.37 (0.34-0.39) | <0.001 | 1316 (35.4) | 0.53 (0.49-0.57) | <0.001 |
| **Malignant** **tumor** |  |  |  |  |  |  |
| No | 18453 (67.9) |  |  | 9024 (48.1) |  |  |
| Yes | 178 (49.3) | 0.46 (0.37-0.57) | <0.001 | 123 (39.9) | 0.72 (0.57-0.90) | 0.005 |
| **Neurological disorder** | |  |  |  |  |  |
| No | 18396 (67.8) |  |  | 9019 (48.0) |  |  |
| Yes | 235 (59.6) | 0.70 (0.57-0.86) | <0.001 | 128 (43.1) | 0.82 (0.65-1.03) | 0.092 |
| **Psychological disorder** | |  |  |  |  |  |
| No | 17756 (66.9) |  |  | 8736 (47.3) |  |  |
| Yes | 875 (86.7) | 3.23 (2.70-3.89) | <0.001 | 411 (68.0) | 2.37 (2.00-2.83) | <0.001 |
| **Renal diseases** | |  |  |  |  |  |
| No | 18473 (67.9) |  |  | 9045 (48.1) |  |  |
| Yes | 18631 (67.7) | 0.41 (0.33-0.51) | <0.001 | 102 (36.0) | 0.61 (0.47-0.77) | <0.001 |
| **Respiratory disease** |  |  |  |  |  |  |
| No | 17045 (67.5) |  |  | 8258 (47.5) |  |  |
| Yes | 1586 (69.0) | 1.07 (0.98-1.17) | 0.153 | 889 (52.2) | 1.21 (1.09-1.33) | <0.001 |
| **Suppressed immune function** | |  |  |  |  |  |
| No | 18244 (67.5) |  |  | 8939 (47.8) |  |  |
| Yes | 387 (73.9) | 1.36 (1.12-1.66) | 0.002 | 208 (53.6) | 1.26 (1.03-1.54) | <0.025 |
| **Other comorbidity** |  |  |  |  |  |  |
| No | 16340 (67.2) |  |  | 7907 (47.4) |  |  |
| Yes | 2291 (71.0) | 1.19 (1.10-1.29) | <0.001 | 1240 (52.0) | 1.20 (1.10-1.31) | <0.001 |

AEFI: adverse event following immunization. OR: odds ratio. BMI: body mass index. Conf.: PCR-confirmed

**Table S6.** Univariate logistic regression of factors potentially associated with the occurrence of a local reaction

|  | **Dose 1** |  |  | **Dose 2** |  |  |
| --- | --- | --- | --- | --- | --- | --- |
| Local reaction | Reported ≥1 local AEFI (%) | Crude OR (95%CI) | p-value | Reported ≥1 local AEFI (%) | Crude OR (95%CI) | p-value |
| **Sex** |  |  |  |  |  |  |
| Males | 2729 (25.7) |  |  | 1392 (16.7) |  |  |
| Females | 8878 (52.4) | 3.18 (3.02-3.36) | <0.001 | 3451 (32.1) | 2.35 (2.19-2.52) | <0.001 |
| **Age** |  |  |  |  |  |  |
| <40 years | 3567 (56.6) |  |  | 1309 (39.8) |  |  |
| 40-54 years | 3799 (54.9) | 0.93 (0.87-1.00) | 0.051 | 1378 (37.2) | 0.90 (0.81-0.99) | 0.026 |
| 55-74 years | 2964 (42.0) | 0.55 (0.52-0.59) | <0.001 | 1298 (23.3) | 0.46 (0.42-0.51) | <0.001 |
| ≥75 years | 1277 (17.6) | 0.16 (0.15-0.18) | <0.001 | 858 (13.2) | 0.23 (0.21-0.25) | <0.001 |
| **Vaccine** |  |  |  |  |  |  |
| AstraZeneca | 4991 (56.9) |  |  | 1355 (24.4) |  |  |
| Johnson&Johnson | 980 (39.9) | 0.50 (0.46-0.55) | <0.001 | - | - | - |
| Moderna | 2066 (60.3) | 1.15 (1.06-1.25) | <0.001 | 1378 (53.0) | 3.49 (3.16-3.85) | <0.001 |
| Pfizer | 3570 (27.7) | 0.29 (0.27-0.31) | <0.001 | 2110 (19.3) | 0.74 (0.68-0.80) | <0.001 |
| **BMI** |  |  |  |  |  |  |
| <18.5 | 203 (49..5) |  |  | 89 (33.8) |  |  |
| 18.5-24.9 | 5880 (42.9) | 0.77 (0.63-0.93) | 0.008 | 2528 (26.9) | 0.72 (0.56-0.94) | 0.013 |
| 25.0-29.9 | 3577 (38.9) | 0.65 (0.53-0.79) | <0.001 | 1501 (22.9) | 0.58 (0.45-0.76) | <0.001 |
| ≥30.0 | 1823 (46.5) | 0.89 (0.72-1.09) | 0.246 | 678 (25.5) | 0.67 (0.51-0.88) | 0.004 |
| Unknown | 124 (42.8) | 0.76 (0.56-1.03) | 0.078 | 47 (25.4) | 0.67 (0.44-1.01) | 0.057 |
| **Conf. COVID-19 infection** | |  |  |  |  |  |
| No | 10691 (41.2) |  |  | 4518 (25.0) |  |  |
| Yes | 916 (58.3) | 2.00 (1.80-2.12) | <0.001 | 325 (33.4) | 1.51 (1.32-1.73) | <0.001 |
| **Use of antipyretic drug** | |  |  |  |  |  |
| No | 9609 (40.5) |  |  | - | - | - |
| Yes | 1991 (52.7) | 1.64 (1.53-1.76) | <0.001 | - | - | - |
| **Any comorbidity** |  |  |  |  |  |  |
| No | 6323 (44.3) |  |  | 2403 (26.2) |  |  |
| Yes | 5284 (39.8) | 0.83 (0.79-0.87) | <0.001 | 2440 (24.6) | 0.92 (0.86-0.98) | 0.011 |
| **Allergy** |  |  |  |  |  |  |
| No | 9968 (40.7) |  |  | 4144 (24.2) |  |  |
| Yes | 1639 (54.1) | 1.72 (1.59-1.85) | <0.001 | 699 (35.3) | 1.71 (1.55-1.88) | <0.001 |
| **Cardiovascular disorder** | |  |  |  |  |  |
| No | 10901 (43.8) |  |  | 4463 (26.6) |  |  |
| Yes | 706 (26.5) | 0.46 (0.42-0.51) | <0.001 | 380 (16.7) | 0.55 (0.49-0.62) | <0.001 |
| **Diabetes** |  |  |  |  |  |  |
| No | 11241 (42.8) |  |  | 4657 (25.9) |  |  |
| Yes | 366 (28.4) | 0.53 (0.47-0.60) | <0.001 | 186 (17.4) | 0.60 (0.51-0.71) | <0.001 |
| **Hepatic disease** | |  |  |  |  |  |
| No | 11572 (42.1) |  |  | 4828 (25.4) |  |  |
| Yes | 35 (50.7) | 1.41 (0.88-2.27) | 0.151 | 15 (31.2) | 1.34 (0.71-2.42) | 0.352 |
| **Hypertension** |  |  |  |  |  |  |
| No | 10292 (44.7) |  |  | 4178 (27.2) |  |  |
| Yes | 1315 (29.1) | 0.51 (0.47-0.54) | <0.001 | 665 (17.9) | 0.58 (0.53-0.64) | <0.001 |
| **Malignant tumor** | |  |  |  |  |  |
| No | 11492 (42.3) |  |  | 4775 (25.4) |  |  |
| Yes | 115 (31.9) | 0.64 (0.51-0.80) | <0.001 | 68 (22.1) | 0.83 (0.63-1.08) | 0.179 |
| **Neurological disorder** | |  |  |  |  |  |
| No | 11464 (42.2) |  |  | 4773 (25.4) |  |  |
| Yes | 143 (36.3) | 0.78 (0.63-0.96) | 0.018 | 70 (23.6) | 0.90 (0.69-1.18) | 0.468 |
| **Psychological disorder** | |  |  |  |  |  |
| No | 11024 (41.6) |  |  | 4597 (24.9) |  |  |
| Yes | 583 (57.8) | 1.93 (1.70-2.19) | <0.001 | 246 (40.7) | 2.07 (1.76-2.45) | <0.001 |
| **Renal disease** | |  |  |  |  |  |
| No | 11516 (42.3) |  |  | 4797 (25.5) |  |  |
| Yes | 91 (26.7) | 0.50 (0.39-0.62) | <0.001 | 46 (16.3) | 0.57 (0.41-0.77) | <0.001 |
| **Respiratory disease** | |  |  |  |  |  |
| No | 10600 (42.0) |  |  | 4371 (25.2) |  |  |
| Yes | 1007 (43.8) | 1.08 (0.99-1.17) | 0.093 | 472 (27.7) | 1.14 (1.02-1.27) | 0.021 |
| **Suppressed immune function** | |  |  |  |  |  |
| No | 11351 (42.0) |  |  | 4724 (25.3) |  |  |
| Yes | 256 (48.9) | 1.32 (1.11-1.57) | 0.002 | 119 (30.7) | 1.31 (1.05-1.62) | 0.016 |
| **Other comorbidity** | |  |  |  |  |  |
| No | 10102 (41.6) |  |  | 4111 (24.6) |  |  |
| Yes | 1505 (46.6) | 1.23 (1.14-1.32) | <0.001 | 732 (30.7) | 1.35 (1.23-1.49) | <0.001 |

AEFI: adverse event following immunization. OR: odds ratio. BMI: body mass index. Conf.: PCR-confirmed

**
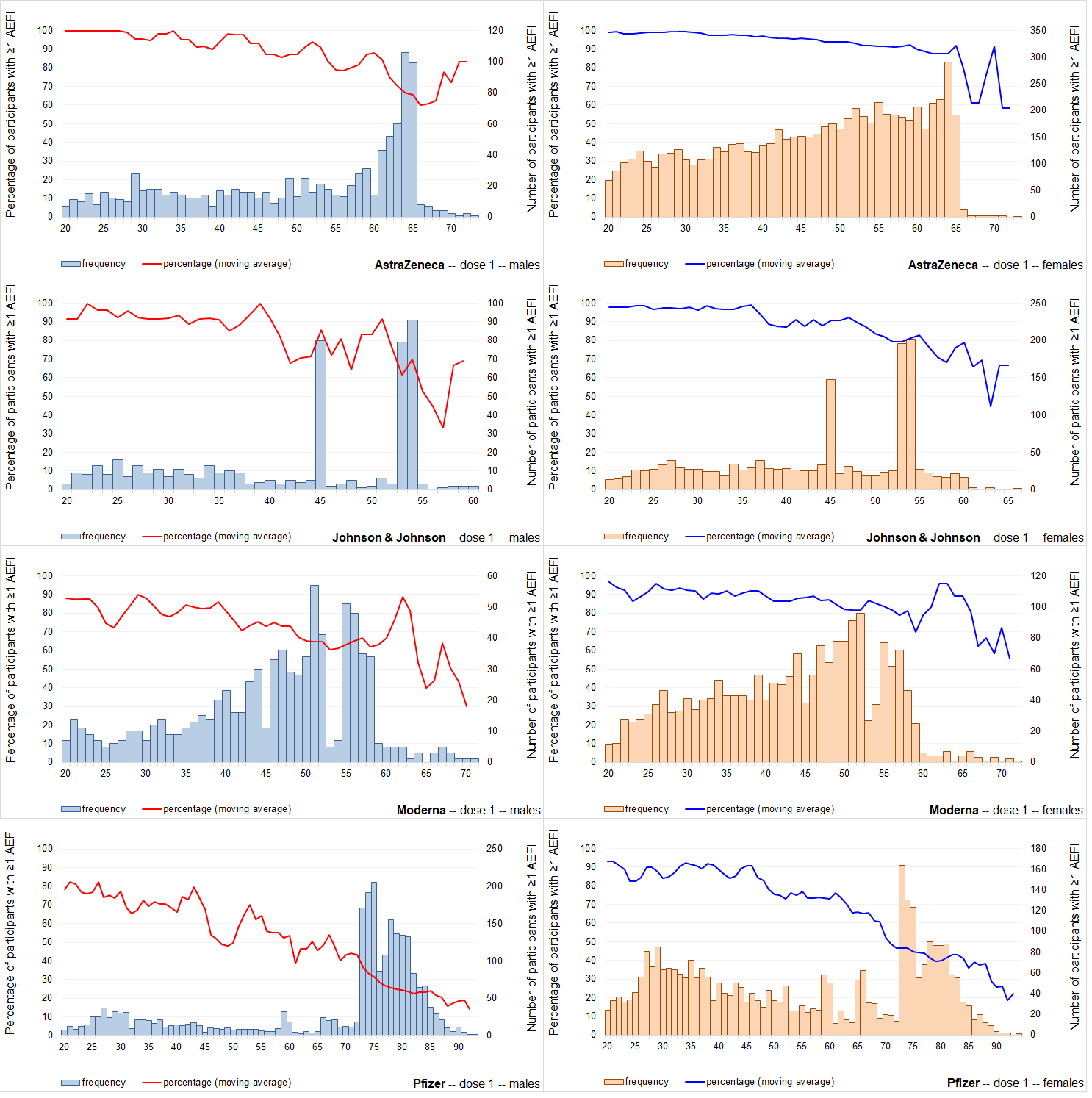

Figure S1.** Number and percentage of male and female vaccinees who reported at least one adverse event after immunization (AEFI) after the first dose of vaccination for each vaccine brand.

**Table S7.** Median and interquartile range of the time to onset and time to recovery of AEFIs by sex, dose and vaccine.

|  | **Dose 1** | | | | | | **Dose 2** | | | | | |
| --- | --- | --- | --- | --- | --- | --- | --- | --- | --- | --- | --- | --- |
|  | **Time to onset (hours)** | | | **Time to recovery (hours)** | | | **Time to onset (hours)** | | | **Time to recovery (hours)** | | |
| **AEFI** | **Males  median  (IQR)** | **Females  median  (IQR)** | **Sign.** | **Males  median  (IQR)** | **Females  median  (IQR)** | **Sign.** | **Males  median  (IQR)** | **Females  median  (IQR)** | **Sign.** | **Males  median  (IQR)** | **Females  median  (IQR)** | **Sign.** |
| **Any AEFI** |  |  |  |  |  |  |  |  |  |  |  |  |
| AstraZeneca | 7 (3-10) | 6 (2-9) | yes | 48 (24-72) | 48 (24-96) | yes | 4 (1-8) | 4 (1-8) |  | 30 (24-72) | 48 (24-72) |  |
| Johnson&Johnson | 7 (3-12) | 5 (1-8) |  | 24 (24-72) | 48 (24-96) | yes |  |  |  |  |  |  |
| Moderna | 3 (1-6) | 3 (1-6) |  | 48 (24-72) | 48 (48-72) | yes | 5 (2-10) | 5 (2-8) |  | 28 (24-48) | 48 (24-72) | yes |
| Pfizer | 3 (1-6) | 2 (1-6) |  | 24 (48-72) | 24 (48-72) | yes | 4 (1-8) | 3 (1-7) |  | 24 (48-72) | 48 (24-72) |  |
| All vaccines | 4 (1-8) | 4 (1-8) |  | 48 (24-72) | 48 (24-96) | yes | 4 (1-9) | 4 (1-8) |  | 48 (24-72) | 48 (24-72) | yes |
| **Local reaction** |  |  |  |  |  |  |  |  |  |  |  |  |
| AstraZeneca | 3 (1-10) | 3 (1-8) |  | 72 (48-120) | 96 (72-144) | yes | 1 (1-8) | 2 (1-4) |  | 48 (24-72) | 72 (48-96) | yes |
| Johnson&Johnson | 1 (0-7) | 1 (0-4) |  | 72 (48-120) | 96 (72-144) | yes |  |  |  |  |  |  |
| Moderna | 2 (1-5) | 3 (1-6) |  | 72 (48-72) | 72 (48-96) | yes | 3 (1-6) | 3 (1-6) |  | 48 (48-72) | 72 (48-96) | yes |
| Pfizer | 3 (1-6) | 3 (1-6) |  | 48 (24-72) | 48 (24-72) | yes | 3 (1-6) | 3 (1-6) |  | 48 (24-72) | 48 (24-72) | yes |
| All vaccines | 3 (1-6) | 2 (1-6) |  | 48 (48-72) | 72 (48-120) | yes | 3 (1-6) | 3 (1-5) |  | 48 (24-72) | 72 (48-96) | yes |
| **Inj. site pain** |  |  |  |  |  |  |  |  |  |  |  |  |
| AstraZeneca | 3 (1-11) | 3 (1-8) |  | 72 (48-120) | 96 (72-144) | yes | 1 (1-10) | 2 (1-5) |  | 48 (24-72) | 72 (48-96) | yes |
| Johnson&Johnson | 1 (0-7) | 1 (0-4) |  | 72 (48-120) | 96 (72-144) | yes |  |  |  |  |  |  |
| Moderna | 2 (1-5) | 3 (1-6) |  | 48 (48-72) | 72 (48-96) | yes | 4 (1-6) | 3 (1-6) |  | 48 (48-72) | 72 (48-96) | yes |
| Pfizer | 3 (1-6) | 3 (1-6) |  | 48 (24-72) | 48 (24-72) | yes | 3 (1-6) | 3 (1-6) |  | 48 (24-72) | 48 (24-72) | yes |
| All vaccines | 3 (1-6) | 3 (1-6) |  | 48 (48-72) | 72 (48-120) | yes | 3 (1-6) | 3 (1-6) |  | 48 (24-72) | 48 (24-72) | yes |
| **Fatigue** |  |  |  |  |  |  |  |  |  |  |  |  |
| AstraZeneca | 9 (6-12) | 8 (5-12) |  | 48 (24-96) | 72 (48-144) | yes | 7 (4-12) | 8 (4-12) |  | 48 (24-90) | 48 (24-96) |  |
| Johnson&Johnson | 8 (6-12) | 8 (5-12) |  | 48 (24-72) | 72 (42-120) | yes |  |  |  |  |  |  |
| Moderna | 5 (3-10) | 5 (3-10) |  | 48 (24-72) | 48 (24-96) |  | 8 (5-12) | 8 (5-12) |  | 24 (48-72) | 24 (48-96) | yes |
| Pfizer | 4 (2-12) | 4 (2-9) |  | 48 (24-96) | 72 (24-120) |  | 6 (1-12) | 6 (3-12) |  | 48 (24-96) | 48 (24-96) |  |
| All vaccines | 8 (4-12) | 8 (4-12) |  | 48 (24-96) | 72 (48-120) | yes | 8 (4-12) | 8 (4-12) |  | 48 (24-72) | 48 (24-96) | yes |
| **Myalgia** |  |  |  |  |  |  |  |  |  |  |  |  |
| AstraZeneca | 9 (6-12) | 8 (6-12) |  | 48 (24-72) | 48 (24-72) |  | 7 (3-10) | 8 (3-11) |  | 48 (24-72) | 48 (24-72) |  |
| Johnson&Johnson | 8 (4-12) | 8 (5-12) |  | 48 (24-72) | 48 (24-72) |  |  |  |  |  |  |  |
| Moderna | 4 (2-8) | 4 (2-8) |  | 48 (48-72) | 48 (48-72) |  | 6 (3-11) | 8 (4-12) |  | 48 (24-53) | 48 (24-72) |  |
| Pfizer | 4 (2-7) | 4 (1-8) |  | 48 (24-72) | 48 (24-72) |  | 4 (1-10) | 5 (2-9) |  | 48 (24-72) | 48 (24-72) |  |
| All vaccines | 6 (3-11) | 8 (4-12) |  | 48 (24-72) | 48 (24-72) |  | 5 (2-10) | 6 (3-12) |  | 48 (24-72) | 48 (24-72) |  |
| **Headache** |  |  |  |  |  |  |  |  |  |  |  |  |
| AstraZeneca | 9 (5-12) | 8 (5-12) |  | 36 (24-48) | 48 (24-72) | yes | 8 (4-8) | 7 (3-12) |  | 24 (48-48) | 24 (48-48) |  |
| Johnson&Johnson | 10 (7-12) | 8 (5-12) |  | 24 (24-48) | 48 (24-72) |  |  |  |  |  |  |  |
| Moderna | 6 (2-11) | 6 (2-12) |  | 24 (24-48) | 48 (24-72) | yes | 9 (6-12) | 9 (5-12) |  | 24 (48-48) | 48 (24-72) | yes |
| Pfizer | 4 (2-8) | 5 (1-8) |  | 48 (24-72) | 48 (24-72) |  | 6 (3-12) | 5 (2-12) |  | 24 (48-48) | 48 (24-72) |  |
| All vaccines | 8 (4-12) | 8 (4-12) |  | 24 (24-48) | 48 (24-72) | yes | 8 (4-12) | 7 (3-12) |  | 24 (48-48) | 48 (24-72) | yes |
| **Malaise** |  |  |  |  |  |  |  |  |  |  |  |  |
| AstraZeneca | 8 (6-12) | 8 (6-11) |  | 48 (24-72) | 48 (24-72) | yes | 8 (4-11) | 8 (4-12) |  | 24 (24-48) | 48 (24-72) |  |
| Johnson&Johnson | 9 (6-12) | 7 (5-10) |  | 24 (24-48) | 48 (24-72) | yes |  |  |  |  |  |  |
| Moderna | 6 (3-12) | 6 (3-12) |  | 48 (24-72) | 48 (24-72) | yes | 10 (6-12) | 8 (5-12) |  | 24 (24-48) | 48 (24-72) | yes |
| Pfizer | 4 (1-9) | 4 (1-8) |  | 24 (48-72) | 48 (24-96) | yes | 8 (1-12) | 7 (3-12) |  | 48 (24-72) | 48 (24-72) |  |
| All vaccines | 8 (4-12) | 8 (5-11) |  | 48 (24-72) | 48 (24-72) | yes | 8 (4-12) | 8 (4-12) |  | 24 (24-48) | 48 (24-72) | yes |
| **Chills** |  |  |  |  |  |  |  |  |  |  |  |  |
| AstraZeneca | 10 (8-12) | 8 (7-11) | yes | 24 (12-24) | 24 (12-48) |  | 12 (10-14) | 10 (7-12) |  | 24 (4-24) | 24 (8-24) |  |
| Johnson&Johnson | 10 (7-12) | 9 (7-11) |  | 24 (8-24) | 24 (10-24) |  |  |  |  |  |  |  |
| Moderna | 8 (5-12) | 10 (5-12) |  | 24 (24-36) | 24 (24-48) |  | 12 (8-14) | 10 (7-12) |  | 24 (24-24) | 24 (24-48) |  |
| Pfizer | 3 (1-12) | 5 (2-10) |  | 24 (13,5-48) | 24 (24-48) |  | 12 (7-13) | 10 (5-14) |  | 24 (24-48) | 24 (24-48) |  |
| All vaccines | 10 (7-12) | 8 (6-11) |  | 24 (12-36) | 24 (12-48) |  | 12 (8-14) | 10 (6-12) |  | 24 (24-48) | 24 (24-48) |  |
| **Pyrexia** |  |  |  |  |  |  |  |  |  |  |  |  |
| AstraZeneca | 10 (8-12) | 10 (7-12) |  | 24 (24-48) | 24 (24-48) |  | 8 (6-13) | 9 (7-12) |  | 24 (24-30) | 24 (24-48) |  |
| Johnson&Johnson | 10 (8-12) | 9 (7-12) |  | 24 (22-27) | 24 (24-48) |  |  |  |  |  |  |  |
| Moderna | 9 (6-12) | 11 (8-14) |  | 24 (24-48) | 24 (24-48) |  | 12 (8-12) | 10 (8-12) |  | 24 (24-48) | 24 (24-48) |  |
| Pfizer | 10 (8-12) | 8 (4-14) |  | 24 (24-48) | 24 (24-48) |  | 12 (5-15) | 12 (8-15) |  | 24 (24-48) | 24 (24-48) |  |
| All vaccines | 10 (8-12) | 10 (7-12) |  | 24 (24-48) | 24 (24-48) |  | 11 (8-13) | 10 (8-12) |  | 24 (24-48) | 24 (24-48) |  |
| **Arthralgia** |  |  |  |  |  |  |  |  |  |  |  |  |
| AstraZeneca | 10 (6-12) | 9 (6-12) |  | 48 (24-72) | 48 (24-72) |  | 8 (7-11) | 8 (5-10) |  | 24 (24-48) | 48 (24-72) |  |
| Johnson&Johnson | 10 (6-12) | 8 (6-12) |  | 48 (24-72) | 48 (24-72) |  |  |  |  |  |  |  |
| Moderna | 4 (2-6) | 8 (5-15) |  | 48 (36-72) | 48 (24-72) |  | 9 (7-12) | 10 (6-12) |  | 24 (24-48) | 48 (24-72) |  |
| Pfizer | 8 (3-13) | 7 (2-12) |  | 48 (24-96) | 48 (24-120) |  | 4 (1-12) | 8 (4-12) |  | 48 (24-120) | 48 (24-96) |  |
| All vaccines | 9 (5-12) | 9 (6-12) |  | 48 (24-72) | 48 (24-72) |  | 8 (4-12) | 8 (5-12) |  | 48 (24-72) | 48 (24-72) |  |
| **Nausea** |  |  |  |  |  |  |  |  |  |  |  |  |
| AstraZeneca | 11 (8-14) | 8 (5-12) |  | 24 (24-48) | 24 (24-48) |  | 8 (7-8) | 7 (3-12) |  | 24 (24-72) | 24 (24-48) |  |
| Johnson&Johnson | 10 (7-15) | 9 (6-12) |  | 24 (13,5-48) | 24 (24-48) |  |  |  |  |  |  |  |
| Moderna | 6 (3-8) | 8 (3-15) |  | 48 (24-48) | 48 (24-72) |  | 10 (5-12) | 10 (6-12) |  | 24 (24-48) | 24 (24-48) |  |
| Pfizer | 11 (3-12) | 3 (0-10) |  | 24 (21-72) | 48 (24-72) |  | 3 (1-6) | 8 (2-12) |  | 24 (24-48) | 24 (24-48) |  |
| All vaccines | 10 (5-14) | 8 (4-12) |  | 24 (24-48) | 24 (24-48) |  | 6 (3-11) | 9 (4-12) |  | 24 (24-48) | 24 (24-48) |  |
| **Inj. site inflammation** |  |  |  |  |  |  |  |  |  |  |  |  |
| AstraZeneca | 3 (1-11) | 3 (1-8) |  | 96 (72-144) | 120 (96-168) | yes | 4 (2-6) | 1 (1-5) |  | 48 (30-72) | 72 (48-96) |  |
| Johnson&Johnson | 1 (0-3) | 1 (0-4) |  | 96 (72-126) | 120 (72-168) |  |  |  |  |  |  |  |
| Moderna | 3 (1-6) | 3 (1-6) |  | 48 (72-96) | 72 (48-120) |  | 4 (3-7) | 2 (1-6) |  | 72 (48-96) | 72 (48-96) |  |
| Pfizer | 3 (1-6) | 3 (1-6) |  | 72 (48-72) | 72 (48-96) |  | 3 (1-10) | 2 (1-5) |  | 72 (48-96) | 72 (48-96) |  |
| All vaccines | 3 (1-7) | 3 (1-6) |  | 72 (48-96) | 96 (72-144) | yes | 4 (1-8) | 2 (1-5) |  | 72 (48-96) | 72 (48-96) |  |

AEFI: adverse event following immunization. IQR: interquartile range. Sign.: significant Kruskal-Wallis test with Bonferroni correction. Inj.: injection.


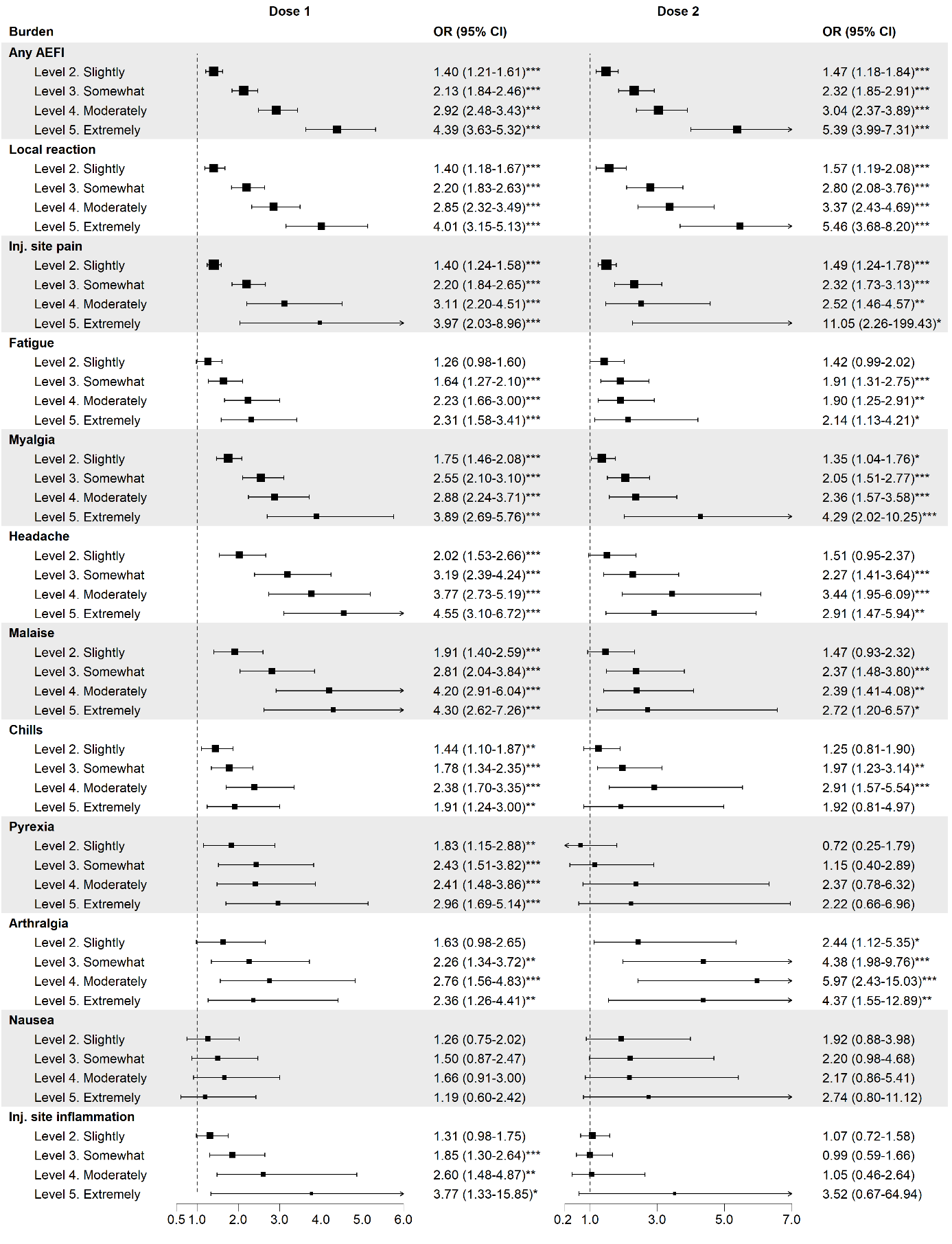

**Figure S2.** Forest plot of the odds of being female in vaccinees who categorized the adverse event following immunization (AEFI) as slightly, somewhat, moderately or extremely burdensome versus not at all burdensome after the first and second dose of vaccination, based on multivariable logistic regression. The size of the square corresponds to the inverse of the standard error.
OR: odds ratio, adjusted for age and vaccine brand. Inj.: injection. * p<0.05, ** p<0.01, *** p<0.001.

Records removed *before screening*:

Duplicate records found

(n = **784**)

Records identified through PubMed search
(n = **1220**)

**Identification**

Records screened

(n = **436**)

Records excluded based on title and/or abstract

(n = **338**)

**Screening**

Full-text records assessed for eligibility
(n=**98**)

Records excluded based on full-text content

(n = **14**)

Records included in review
(n= **84**)

**Included**

**Figure S3.** PRISMA 2020 flow diagram for new systematic reviews which included searches of databases only.

*Adapted from:* Page MJ, McKenzie JE, Bossuyt PM, Boutron I, Hoffmann TC, Mulrow CD, et al. The PRISMA 2020 statement: an updated guideline for reporting systematic reviews. BMJ 2021;372:n71. doi: 10.1136/bmj.n71. For more information, visit: <http://www.prisma-statement.org/>

**Table S8**. Characteristics and main outcomes of epidemiological studies presenting sex-disaggregated outcomes of reported adverse events after COVID-19 vaccination

| **First author, year [reference]** | **Country/ countries** | **Study type** | **Vaccine brand(s)** | **Size of the study population*** | **Main outcomes** |
| --- | --- | --- | --- | --- | --- |
| Adam, 2021 [17] | Saudi Arabia | Cross-sectional study | AstraZeneca, Pfizer | 330 vaccinees (66% males) | Frequency of symptoms was significantly higher in males compared to females for both doses of vaccination (overall: 65.5% *vs.* 34.5%, p: 0.006) |
| Al Bahrani, 2021 [18] | Saudi Arabia | Cross-sectional study (oral questionnaire) | AstraZeneca | 1592 vaccinated persons (81% males) | A higher fraction of males *vs*. females reported: - any reaction (M 76.7% *vs*. F 23.3%, p: 0.001); - fever (M 76.9% *vs*. F 23.1, p: 0.005); - skin rash (M 81.1% *vs*. F 18.9%, p: 0.005); - injection site pain (M 77.3% *vs*. F 22.7%, p: 0.01); - myalgia/joint pain (M 78.1% *vs.* F 21.9%, p<0.0001); - gastrointestinal symptoms (M 82.8% *vs.* F 17.2%, p<0.0001). |
| Al Ghafri, 2021 [19] | Oman | Cross-sectional study (oral questionnaire) | AstraZeneca, Pfizer | 753 vaccinees (54% males) | Significant higher fraction of females *vs*. males reported ≥1 AE (F 54.9% *vs.* M 45.5%, p: 0.01). |
| Al Khames Aga, 2021 [23] | Iraq, Jordan | Cross-sectional study (online and oral questionnaire) | AstraZeneca, Pfizer, Sinopharm | 1736 (52% males) | Mean duration of symptoms was longer in females compared to males for all three vaccine brands though not significant.  Pfizer: mean±SD/day males *vs.* females: 1.52±1.51 *vs.* 1.94±2.29. AstraZeneca: mean±SD/day males *vs.* females: 1.65±2.02 *vs.* 1.49±1.69. |
| Alessa, 2022 [20] | Saudi Arabia | Cross-sectional study (online questionnaire) | AstraZeneca, Pfizer | 612 surgeons (70% males) | A significant association was found between sex and severity of side effects after vaccination. In 53.5% of the female surgeons, the work performance was affected by the side effects compared to 36.8% of male surgeons (p: 0.000). |
| Alghamdi, 2021 [21] | Saudi Arabia | Cross-sectional study (online questionnaire) | AstraZeneca, Pfizer | 4,107 vaccinees (31% males) | Females were more likely to have moderate and severe adverse effects after vaccination as compared to males (rho: -0.133; p<0.015). Also, females significantly reported more local, systemic and allergic reactions compared to males. (Fractions and chi-square tests of the most frequent adverse effects reported by males and females are provided in the article.) |
| Alharbi, 2022 [58] | Saudi Arabia | Prospective cohort study (3-8 months follow-up) | AstraZeneca, Pfizer | 18,543 vaccinees (60% males) | Males were more likely to contract a COVID-19 infection after vaccination than females (M 65.9% *vs.* F 34.1%. aOR 1.17, 95%CI 1.04-1.31, p: 0.009). |
| Aliberti, 2022 [22] | Italy | Cross-sectional study (online questionnaire) | AstraZeneca | 500 vaccinated persons (all professors; 41% males) | A higher percentage of females experienced adverse reactions (55.7% females *vs.* 32.5% males. OR 2.82, 95%CI 1.80-4.42, p<0.001). Also, the perceived health after the 1^st^ and 2^nd^ dose of vaccination was significantly lower for females compared to males. Females reported more adverse reactions with mild/moderate severity compared to males. Significant higher fraction of females reported: - Injection site swelling/pain: OR 2.01, 95%CI 1.31–3.09, p: 0.001; - Fatigue: OR 2.27, 95%CI 1.45–3.56, p<0.001; - Chills: OR 5.56, 95%CI 3.42–9.03, p<0.001; - Dizziness: OR 0.19, 95%CI 0.04–0.87, p: 0.032; - Nausea: OR 1.38, 95%CI 4.06–31.89, p<0.001; - Feeling unwell: OR 1.67, 95%CI 1.04–2.70, p: 0.033. |
| Almohaya, 2021 [24] | Saudi Arabia | Cross-sectional study (online questionnaire) | Pfizer | 3,639 vaccinees (37% males) | A higher fraction of females *vs.* males experienced any adverse reaction (OR adjusted for age: 1.26, 95%CI 1.10-1.44, p: 0.001). Also, a higher fraction of females used anti-pyretic drugs without experiencing an adverse event (OR adjusted for age: 1.54, 95%CI 1.27-1.87, p<0.001). Hospital admission after vaccination was not associated with sex. |
| Almohaya, 2022 [81] | Saudi Arabia | Retrospective cohort study | Pfizer | 71,221 vaccinated individuals (56% males) | A higher fraction of females *vs.* males reported an acute adverse event (OR 1.69, 95%CI 1.21-2.34, p: 0.002). |
| Almufty, 2021 [25] | Iraq | Cross-sectional study (online questionnaire) | AstraZeneca, Pfizer, Sinopharm | 1,012 vaccinees (60% males) | A lower fraction of males *vs.* females experienced an adverse reaction after vaccination (OR 0.67, 95%CI 0.47-0.96, p: 0.028). No difference in severity was observed between males and females. A higher fraction of females compared to males had an elevated D-dimer level, though this was borderline significant. |
| Amodio, 2022 [59] | Italy | Prospective cohort study (online daily questionnaire for 7 days after 1^st^ and 2^nd^ dose) | Pfizer | 293 vaccinees (54% males) | A higher fraction of females *vs.* males experienced adverse events:  - Systemic reaction after 1^st^ dose: crude OR: 1.99; 95%CI 1.11-3.57, p-value not provided.  - Systemic reaction after 2^nd^ dose: adjusted OR: 1.79; 95%CI 1.06-3.02, p-value not provided. |
| Anastassopoulou, 2022 [60] | Greece | Prospective cohort study | Pfizer | 35 vaccinated health care workers (43% males) | Injection site pain after the 1^st^ dose was reported by 46.0% and 30.7% of females and males respectively and after the 2^nd^ dose by 31.5% and 15.3% of females and males respectively, which was a significant difference (1^st^ dose p: 0.002, 2^nd^ dose p<0.001). |
| Azzolini, 2022 [61] | Italy | Prospective cohort study (online questionnaire) | Pfizer | 4,156 vaccinated health care professionals (38% males) | The occurrence of any adverse event was higher in females *vs.* males (OR 1.95; 95%CI 1.74-2.19, p<0.001). |
| Bae, 2021 [62] | South Korea | Prospective cohort (online questionnaire, three days follow-up) | AstraZeneca, Pfizer | 5866 vaccinees (24% males) | For the AstraZeneca vaccine, a higher fraction of females *vs.* males reported adverse reactions (F 95.0% *vs.* M 87.7%, p<0.001). For the Pfizer vaccine, no sex differences were found in reported adverse reactions. (Fractions and chi-square tests of the most frequent adverse effects reported by males and females are provided in the article.) |
| Bauernfeind, 2021 [26] | Germany | Cross-sectional study | Pfizer | 735 vaccinees (26% males) | A higher fraction of females reported an adverse event after vaccination compared to males (1^st^ dose: F 5.3% *vs.* M 1.6%; 2^nd^ dose: F 38.3% *vs.* M 21.0%). The OR for experiencing a severe adverse event was 2.4 (95%CI 1.7-3.6) in females *vs.* males. |
| Beatty, 2021 [63] | United States | Prospective cohort study (online questionnaires) | Johnson& Johnson, Moderna, Pfizer | 46,204 vaccinees (30% males) | A higher portion of females *vs.* males reported an adverse effect (OR 1.65; 95%CI 1.53-1.78, p<0.001). |
| Borroni, 2021 [27] | Italy | Cross-sectional study | Pfizer | 3,659 vaccinated healthcare workers (29% males) | A higher fraction of females *vs.* males reported a systemic adverse reaction (F 59.6% *vs.* M 50.0%. adjusted RR 1.14, 95%CI 1.06-1.24, p-value not provided). |
| Bukhari, 2022 [28] | Saudi Arabia | Cross-sectional  study | AstraZeneca, Pfizer | 1021 vaccinees (29% males) | No statistically significant association between sex and cutaneous vaccine-related side effects was found (p: 0.119) |
| Canlas, 2022 [29] | Oman | Cross-sectional (online questionnaire) | AstraZeneca, Pfizer | 362 respondents | Local and systemic reactogenicity were more common among female recipients (F) of Pfizer vaccine compared to males (M), both after the 1^st^ and 2^nd^ dose: Dose 1: arm pain F 63%, M 47%, p: 0.020%; tiredness F 47%, M 40%, p: 0.017; headache F 34%, M 23%, p: 0.003; muscle pain F 49%,M 37%, p: 0.028; fever F 30%, M 17%, p: 0.017. Dose 2: arm pain F 53%, M 40%, p: 0.011; redness of the arm F 19%, M 17%, p: 0.028; tiredness F 41%, M 30%, p: 0.028; chills F 21%, M 10%, p: 0.036. No association between sex and incidence of side effects found for the AstraZeneca vaccine. |
| Chapin-Bardales, 2021 [64] | United States | Prospective cohort study (online daily questionnaires for 7 days after 1^st^ and 2^nd^ dose) | Moderna, Pfizer | 4,717,908 (35% males) | A higher fraction of females compared to males reported adverse reactions (consistent pattern for dose, vaccine product and reaction type). A higher fraction of females *vs.* males reported a local reaction on day 14 post-vaccination for dose 1 (F 2.0% *vs.* M 0.4%). |
| Coggins, 2021 [65] | United States | Prospective cohort study (questionnaires) | Pfizer | 206 vaccinees (30.6% males) | Females reported more vaccine-related symptoms than males and female sex has been found to be linked to higher symptom scores, although no statistical significance was found. |
| Cohen, 2022 [30] | United States | Cross-sectional study (online questionnaire) | Moderna, Pfizer | 861 respondents (41% males); out of 721 recipients, 192 received one vaccine dose, 529 received both vaccine doses. | Females had a borderline statistically significant likelihood to feel ill enough to miss work and stay home after vaccination (OR 1.61, 95%CI 1.00- 2.58, p: 0.05) compared to men. |
| Costantino, 2022 [31] | Italy | Cross-sectional study (questionnaire) | AstraZeneca, Pfizer | 1788 vaccinees (46% males), including 1613 who received the Pfizer vaccine (45% males) and 175 who received the AstraZeneca vaccine (51% males). | A statistically significant (p: 0.01) higher portion of females reported AEFIs after the 1^st^ Pfizer dose (F 26.2% *vs.* M 15.8%), also accompanied with a higher mean duration (33.9±37.8 (SD) hours *vs.* 29.6±25.3 (SD) hours, p: 0.107), compared to males. The most frequently reported adverse event following the 1^st^ dose of Pfizer vaccine was pain at the injection site among both sexes, followed by asthenia, fatigue, and exhaustion. A higher incidence of pain at the injection site was observed among females *vs.* males (F 17.1% *vs.* M 9.6%). No significant sex-related difference concerning both the incidence (p: 0.697) and the duration of AEFIs (p: 0.343) was observed after the 1^st^ AstraZeneca dose. |
| Cuschieri, 2021 [32] | Malta | Cross-sectional (online questionnaire) | Pfizer | Out of 4885 vaccinated healthcare workers, 1480 responded (33% males) | Compared to males, female participants experienced a higher rate of adverse effects after vaccination, mostly mild to moderate in severity: - pain at injection site (OR 1.91, 95%CI 1.34-2.72, p<0.01); - redness at injection site (OR 2.49, 95%CI 1.66-3.73, p<0.01); - swelling at injection site (OR 1.89, 95%CI 1.33-2.69, p<0.01); - fever (OR 1.74, 95%CI 1.36-2.23, p<0.01);  - chills (OR 2.32, 95%CI 1.83-2.94, p<0.01);  - fatigue (OR 2.43, 95%CI 1.89-3.122, p<0.01); - muscle pain (OR 1.54, 95%CI 1.23-1.94, p<0.01); - joint pains (OR 2.01, 95%CI 1.61-2.69, p<0.01); - headache (OR 2.07, 95%CI 1.63-2.63, p<0.01); - vomiting (OR 3.43, 95%CI 1.18-9.84, p: 0.02), with results adjusted for age and healthcare worker role. |
| Dar-Odeh, 2022 [33] | Saudi Arabia, Jordan | Cross-sectional study (online questionnaire) | AstraZeneca, Pfizer, Sinopharm | 498 vaccinated practitioners (physicians and dentists) (30.1% males). | Males (n=150) reported 21 long-term adverse events (LTAEs) (14.0%), whereas females (n=348) reported 59 (17.0%) LTAEs (p: 0.410) |
| Darraj, 2022 [66] | Saudi Arabia | Prospective cohort study (online questionnaire) | AstraZeneca | 437 healthcare workers who received their 1^st^ vaccine dose were included in the study (35% males) | The percentage of females who reported a side effect was higher than the percentage of males, in particular of dizziness (11.9% *vs.* 3.3%, χ2 5.24, p: 0.022) and chills (F 6.2% *vs.* M 0.0%, χ2 5.86, p: 0.015). In contrast to this, the male healthcare employees had a higher frequency of whole-body pain/fatigue (67.8%) compared to females (50.0%) (χ2 7.40, p: 0.007). Overall, there was no significant association between sex and the probability of reporting a side effect (OR 0.93, 95%CI 0.62-1.38, p: 0.701; males are the reference group). Females demonstrated to be less likely to report a higher number of different side-effects than males (aOR 0.61, 95%CI 0.38-0.97, p: 0.038). Onset of the side effect at day of vaccination in 93.3% of the males and 93.8% of females. Duration of symptoms 2-3 days in 51.1% of the males, 54.4% of the females. Both time to onset and duration not significantly different between males and females. |
| Elnaem, 2021 [34] | Malaysia | Cross-sectional study (online questionnaire) | AstraZeneca, Pfizer, Sinovac | 428 vaccinees aged ≥18 years (33.6% males).Out of 428 respondents, 332 (78%) were vaccinated: 54% received Pfizer vaccine, 27% Sinovac vaccine, 19% AstraZeneca vaccine. | Males (OR 0.51, 95%CI 0.28-0.94, p: 0.030,) and those who received the Sinovac vaccine (OR 0.08, 95%CI 0.03-0.23) had a lower incidence of vaccine-related side effects. |
| El-Shitany, 2021 [35] | Saudi Arabia | Cross-sectional study (online questionnaire) | Pfizer | 455 vaccinees (36% males) | A higher number of female participants (58%) reported different side effects after the vaccination compared to males (28%) (significant difference between the number of males and females, p<0.001). Additionally, the number of females who experienced no side effects (6.2%) was lower than males (7.7%). |
| Filippatos, 2021 [67] | Greece | Prospective cohort study | Pfizer | 502 vaccinated HCWs (males (21.7% males) | No association between sex and local AEs was recorded both after the 1^st^ and the 2^nd^ doses (no statistical significance was found), whereas there were differences in side effects according to sex regarding systemic AEs, especially with fatigue and headache following both vaccine doses. Compared to males, females were more likely to develop: - Fatigue: OR 2.61, 95%CI 1.14-5.95 (dose 1), OR 2.06, 95%CI 1.26-3.35 (dose 2); - Headache: OR 2.57, 95%CI 1.13–5.86 (dose 1), OR 2.73, 95%CI 1.53-4.86 (dose 2). Furthermore, female sex was associated with increased ORs for experiencing myalgias-arthralgias (OR 3.08, 95%CI 1.73–5.49) and chills (OR 3.59, 95%CI 1.06–12.24) after the 2^nd^ dose. |
| Gan, 2022 [36] | Malaysia | Cross-sectional study (online questionnaire) | Pfizer | 2282 vaccinees (24% males) | AEFIs were referred from 1472 (64.5%) vaccinees (19.8% males) (p<0.01). The AEFIs reporting rate was higher among female participants than among male participants: 80.2% (females experiencing adverse events) *vs.* 69.5% (females not experiencing adverse events), compared to 19.8% (males experiencing adverse events) *vs.* 30.5% (males not experiencing adverse events). |
| Ganesan, 2022 [37] | United Arab  Emirates (UAE) | Cross-sectional study (online and oral questionnaires) | Pfizer,  Sinopharm, | Out of 1,878 vaccinees (39% males), 1,134  were telephonically interviewed and 744 completed the online survey. | No statistically significant association between the reactogenicity profile and sex was detected among Pfizer recipients (p: 0.562), in contrast to association with female sex found among Sinopharm recipients. |
| Green, 2022 [12] | Israel | Three cross-sectional studies: national data obtained from spontaneous reports to the Israel Ministry of Health database, a website panel survey, two surveys from a single company | Pfizer | 923 internet panel survey participants (45% males);  266 participants of the first workplace survey following the 1^st^ and 2^nd^ vaccine doses (57% males);  294 participants of the second workplace survey following the 3^rd^ vaccine dose (59% males) | The results of the three different data sources were mostly consistent. Females in all age groups had an increased risk for experiencing any side effect spontaneously reported (local and systemic reactions). The female-to-male risk ratios (F:M RRs) were higher for allergic reactions and after the 2^nd^ dose. As for sex-related differences in AEFIs recorded in the online panel survey, a higher risk for adverse outcomes among females has been shown than among males. The F:M RRs for the 1^st^, 2^nd^ and 3^rd^ doses for AEFIs were higher among female individuals in all age groups. The F:M RRs of reporting any AEFI were fairly consistent:  - Dose 1, F:M RR 1.89, 95%CI 1.37-2.61, p<0.0001;  - Dose 2, F:M RR 1.82, 95%CI 1.45-2.28, p<0.0001).  No clear sex-based differences in seeking medical care:  - Dose 1, F:M RR 1.03, 95%CI 0.60-1.75, p: 0.9259;  - Dose 2, F:M RR 1.13, 95% CI 0.74-1.74, p: 0.5647. Positive association for the occurrence local and systemic reactions: - Pain injected hand: F:M RRs 7.03 (dose 1), 4.13 (dose 2), 4.23 (dose 3); - Rash/redness injection site: F:M RRs 3.71 (dose 1), 5.00 (dose 2), 3.52 (dose 3); - General weakness: F:M RRs 30.67 (dose 1), 2.60 (dose 2), 1.66 (dose 3); - Headache: F:M RRs 9.15 (dose 1), 3.28 (dose 2), 2.45 (dose 3);  - Muscle pains: F:M RRs 5.06 (dose 1), 2.61 (dose 2), 3.25 (dose 3); - Difficulty in standing: F:M RRs 23.86 (dose 2), 1.78 (dose 3). Additionally, the frequency of paresthesia in the injected hand and facial paralysis was higher in females. The reporting rate of AEFIs lasting longer than 24h was higher among females. |
| Hatmal, 2022 [38] | Jordan, Lebanon, Palestine, Saudi Arabia, Qatar, Algeria, Tunisia, Syria, Egypt, Bahrain, Iraq, Libya, Kuwait, Morocco, UAE, Oman, Sudan, Mauritania, Yemen | Cross-sectional study (online questionnaire) | AstraZeneca, Johnson& Johnson, Moderna, Pfizer, Sinopharm, Sputnik V, SinoVac | 10,064 vaccinees (44% males) | According to chi-square tests, sex is a significant predictor (p<0.01) of certain local and systemic side effects, such as tiredness (χ2 348.81), headache (χ2 243.11), pain and swelling at the injection site (χ2 387.31), joint pain (χ2 187.85), myalgia (χ2 179.21), nausea (χ2 274.59), dizziness (χ2 285.06), sleepiness and laziness (χ2 284.72), as well as severity of vaccine-associated side effects (χ2 345.78). No sex-related difference (p>0.01) was detected using chi-square tests for bleeding gums (χ2 1.50) and nose bleeding (χ2 0.00) rate. Similar results regarding the best-predicted side effects based on sex were found using gradient boosting and generalized linear models/surrogate random forest. |
| Hibino, 2021 [82] | Japan | Retrospective cross-sectional study | Moderna | 1098 vaccinees (50% males) | A significant (p<0.001) higher prevalence of injection site reactions after the 1^st^ dose was found in females (F 12.5% *vs.* M 1.5%). Out of 31 cases of reported erythema at the injection site, 28 were females. |
| Higashino, 2022 [83] | Japan | Retrospective cross-sectional study | Moderna | 5893 vaccinees (56% males) | The incidence rate of a delayed-onset adverse skin reaction after the 1^st^ dose was higher among females than males (OR 5.30, 95%CI 4.42-6.34) and among respondents aged 30-69 years. The mean (SD) time to onset was significantly lower for males [6.97 (1.26) days] than for females [7.32 (1.44) days], as well as the mean (SD) duration of adverse reactions was less among males (4.83 [3.27] days) than among females (5.98 [4.43] days). |
| Hoffmann, 2021 [84] | Germany | Retrospective cohort study | Pfizer | 1065 vaccinees (41% males) | A significant higher portion of the local post-vaccination and systemic reactions were reported by females (local: 178/274, p: 0.032; systemic: 95/135, p: 0.012). 64% of females and 73% of males included in the study reported no adverse effects. |
| Izumo, 2021 [68] | Japan | Prospective cohort study (online questionnaires) | Pfizer | 983 (24% males) and 798 (22 % males) healthcare employees who received the 1^st^ and 2^nd^ dose respectively.  23 (57% males) people without history of clinical COVID-19 infection participated in the survey of the antibody titer change after Pfizer vaccination. | Female participants had a significantly higher incidence of side effects than male participants:  - Dose 1: OR 1.88; 95%CI 1.35-2.60, p<0.001;  - Dose 2: OR 2.75; 95%CI 1.71-4.42, p < 0.001.  The 2^nd^ dose elicited significantly more side effects than the 1^st^ dose. The reporting rate of grade 3 side effects (fever with a body temperature of 38°C) was higher among females than males:  - Dose 1: 18.1% *vs.* 12.4%, OR 1.84; 95%CI 1.11-3.03, p: 0.015;  - Dose 2: 51.9% *vs.* 39.9%, OR 1.63; 95%CI 1.16-2.29, p: 0.005.  General fatigue, muscle pain and pain at the injection site were the most frequently reported adverse events and more common in females (dose 1: 29.4%, 50.3%, 65.8%, respectively; dose 2: 71.1%, 53.4%, 69.2%, respectively) than males (dose 1: 13.5%, 16.0%, 24.9%, respectively; dose 2: 57.3%, 47.2%, 60.1%, respectively). No sex-disaggregated data were reported related to changing in antibody level after vaccination. |
| Jacobson, 2021 [69] | United States | Prospective cohort study (online and oral questionnaires) | Moderna, Pfizer | 5567 vaccinated hospital workers, with 1955 recipients of Moderna 1^st^ dose (35% males) and 3612 recipients of Pfizer 1^st^ dose (35% males). Numbers of vaccinees who received the 2^nd^ dose were not provided. | AEFIs occurred only among female personnel and most frequently after the 1^st^ dose: 13 workers who received the Moderna vaccine reported a delayed skin reaction which started 3–9 (median 7) days after their vaccination. Erythema, pain, swelling and pruritus were the most commonly reported adverse effects The overall rate of reporting a delayed injection site reaction to either the 1^st^ or 2^nd^ Moderna vaccine was 1.1% (95%CI 0.6-1.8). The incidence was higher among females aged 31–45 (incidence 2.0%; 95%CI 0.99-3.51%). Only 5/13 female employees had a delayed reaction after the 2^nd^ dose, and the risk of another adverse event after the 2^nd^ dose 2.10 times higher in workers receiving the 2^nd^ injection in the same arm as their 1^st^ injection (95%CI: 0.53-8.29). Male employees did not experience any of reported delayed local injection site reactions (incidence of delayed injection site reactions among Moderna recipients was 0% among males compared to 1.1% among females; p: 0.004). None of the reported AEFIs were associated with Pfizer vaccination. |
| Kang, 2021 [39] | South Korea | Cross-sectional study (online questionnaire) | Pfizer | 131 healthcare workers who received the 1^st^ and 2^nd^ vaccine dose (43% males) | No sex-related differences were observed in severity and incidence of AEFIs following each vaccine dose. |
| Kant, 2022 [5] | The Netherlands | Prospective cohort study (online questionnaires) | AstraZeneca, Johnson& Johnson, Moderna, Pfizer | 27,554 vaccinees completed the survey after the 1^st^ dose. 20,682 vaccinees provided data related to the full vaccination schedule. 8782 (15% males), 2458 (28% males), 3426 (34% males), 12,888 (58% males) individuals received the AstraZeneca, Johnson& Johnson, Moderna and Pfizer vaccine respectively. | The study demonstrated higher incidences of at least one of the well-known systemic adverse reactions among females and in younger individuals. As for both male and female respondents who had an history of COVID-19 infection, an increased systemic reactogenicity after the 1^st^ dose compared with the 2^nd^ dose was observed, regardless of the vaccine brand. |
| Kaur, 2021 [89] | WHO program’s member countries for International Drug Monitoring in VigiBase | Study based on World Health Organization (WHO) database | AstraZeneca, Moderna, Pfizer | Out of 103,954 adverse events reported in the VigiBase database, a total of 4863 cardiovascular adverse events were notified | Significant positive associations between female sex and certain cardiovascular AEFIs, in particular increased blood pressure (OR 2.1, 95%CI 1.8-2.3), supraventricular tachycardia (OR 2.5, 95%CI 1.7-3.8), palpitations (OR 2.1, 95%CI 1.9-2.2). Significant positive association between male sex and some cardiovascular AEFIs, such as sinus tachycardia (OR 4.7, 95%CI 2.8-7.7) and palpitations (OR 1.7; 95%CI 1.4-2.1). Post-vaccination hypertension and severe hypertension were indifferently associated with sexes. |
| Khan, 2022 [40] | Bangladesh | Cross-sectional study (oral questionnaire) | AstraZeneca | 293 vaccinees (46% males) | 74.1% of the vaccinees suffered from side effects after the 1^st^ vaccine dose, with no statistically significant difference by sex (p: 0.109). 55.3% of the vaccinees experienced side effects after the 2^nd^ vaccine dose with a statistically significant predominance in females (F 64.8 % *vs.* M 44.0%, p<0.001) |
| Kitagawa, 2022 [41] | Japan | Cross-sectional (online questionnaire) | Moderna, Pfizer | 7360 individuals who received the 1^st^ dose of Pfizer (n=890) and Moderna (n=6401) (36% and 54% males respectively). 4854 individuals who received the 2^nd^ dose of Pfizer (n=853) and Moderna (n=3965) (38% and 53% males respectively). | A significant higher incidence of nearly all systemic adverse reactions was observed in females compared to males (p-values <0.001), especially in older people (≥50 years), and after the 2^nd^ dose. No significant association between sex and the occurrence of diarrhea (p: 0.26) and skin rash (p: 0.97) among participants vaccinated with Pfizer. A borderline significant association between female sex and frequency of diarrhea (p: 0.047) was observed among participants vaccinated with Moderna. A significant association between sex and local reactions (injection site pain) after the 2^nd^ dose (Pfizer: F 90.7% *vs.* M 86.5%, p: 0.032; Moderna: F 94.1% *vs.* M 92.1%, p: 0.003). The most frequently reported systemic adverse reaction was fatigue among individuals vaccinated with 2^nd^ doses of either vaccine, with a predominance in females (Pfizer: 80.6% females *vs.* 66.9% males; Moderna: F 85.3% *vs.* M 77.7%). |
| Klugar, 2021 [42] | Germany | Cross-sectional study (online questionnaire) | AstraZeneca, Moderna, Pfizer | 599 vaccinated healthcare workers (27% males), including 474 (26% males) who received the Pfizer or Moderna vaccine and 125 (33% males) who received the AstraZeneca vaccine. | Female sex was positively associated with an increased risk of side effects for all three vaccine brands. Female participants who received the AstraZeneca vaccine had a statistically significant higher prevalence of local and systemic side effects compared to males (local OR 2.68, 95%CI 1.20-5.97; systemic OR 3.09, 95%CI 1.06–9.02). No significant association between sex and occurrence of local and systemic side effects for the Pfizer and Moderna vaccines (local OR 1.02, 95%CI 0.62-1.68; systemic OR 1.34, 95%CI 0.88-2.03). Incidence in females compared to males was higher for fever (F 10.6% *vs.* M 7.4%), chills (F 14.9% *vs.* M 11.5%), headache/fatigue (F 51.3% *vs.* M 38.5%), muscle pain (F 28.4% *vs.* M 27%), joint pain (F 15.8% *vs.* M 9.8%), and lymphadenopathy (F 10.3% *vs.* M 4.9%). The skin related side effects (n=13, 3.7%) were exclusively reported by females, in the Pfizer and Moderna vaccine group.  Among AstraZeneca vaccine recipients, females had higher rate of fever (F 53.6% *vs.* 36.6%), chills (F 60.7% *vs.* M 51.2%), headache/fatigue (F 81% *vs.* M 61%), muscle pain (F 56% *vs.* M 46.3%), joint pain (F 52.4% *vs.* M 36.6%), nausea (F 22.6% *vs.* M 17.1%), and malaise (F 51.2% *vs.* M 43.9%) than males. |
| Koh, 2021 [70] | Singapore | Prospective cohort study | Moderna, Pfizer | 1,398,074 vaccinated individuals (55% males) | Out of 1,398,074 vaccinated individuals, 457 (0.03%) experienced a spectrum of neurological disorders (61.5% were males) among which 95.8% received the Pfizer vaccine and 4.2% the Moderna vaccine. Out of 35/73 cases of Central Nervous System Syndromes were male, 197/286 cases of cerebrovascular disorders were male, 33/59 cases of Peripheral Nervous System disorders were male, 16/39 Immunization Stress Related Response were male. |
| Lee, 2021 [71] | South Korea | Prospective cohort study | AstraZeneca | 1603 health care workers (21% males) | Age, sex, BMI identified as risk factors for severe and potentially life threatening AE (grade 3 and grade 4). Risk of developing severe or life threatening AE was higher in females compared to males for all AE (aOR 2.16, 95%CI 1.62-2.89), local reactions (aOR 2.71, 95%CI 1.91-3.93) and systemic reactions (aOR 1.94, 95%CI 1.42-2.70). Diabetes mellitus was a risk factor for developing severe AE in females (aOR 5.50, 95%CI 1.81-20.73) and allergies (not asthma) and hypertension in males (aOR 9.73, 95% CI 1.07-210.61 and aOR 4.02, 95%CI 1.15-14.50 respectively). |
| Lee, 2021 [72] | South Korea | Prospective cohort study (online questionnaires) | Pfizer | 265 healthcare workers (36% males) who received the 2^nd^ vaccine dose | Adverse reactions were more commonly reported among females than among males (F 95.3% *vs.* M 77.9%, p<0.001). A significant higher incidence in females of both local reactions (including pain [F 87.8% *vs.* M 66.3%], redness [F 12.9% *vs.* M 4.2%], and swelling [F 14.7% *vs.* M 2.1%]) and systemic reactions (including fever [F 37.6% *vs.* M 22.1%], chills [F 50.0% *vs.* M 33.7%], muscle ache [F 74.7% *vs.* M 58.9%], joint pain [F 35.9% *vs.* 21.1%], headache [F 54.7% *vs.* M 37.9], and dizziness [F 32.4 *vs.* M 14.7%]) was recorded. |
| Loosen, 2022 [85] | Germany | Retrospective cohort study | AstraZeneca, Moderna, Pfizer | 531,468 vaccinees who received a total of 908,869 COVID-19 vaccinations (51% males) | Male sex was associated with a higher OR for experiencing vaccine-related side effects (OR 1.17, 99%CI 1.14-1.21, p<0.001) |
| Lounis, 2022 [43] | Algeria | Cross-sectional study (questionnaire) | Johnson& Johnson CoronaVac, Sputnik V | 721 vaccinated healthcare workers (41% males) | Female participants showed an overall higher incidence of post-vaccination AE, both local (58.5%) and systemic (59.4%), compared to their male counterparts (35.6% for local AE, 45.8% for systemic AE). For the Johnson & Johnson vaccine, the aOR for experiencing an AE in females *vs.* males was 2.50, 95%CI 1.22-5.51, p: 0.013. |
| Mahallawi, 2021 [44] | Saudi Arabia | Cross-sectional study (questionnaire) | AstraZeneca, Pfizer | 365 respondents (84% males) | The mean total score of vaccine-related symptoms was higher among female *vs.* male participants, in particular after receiving the AstraZeneca vaccine (1.61±1.17 and 1.17±1.09, respectively, p: 0.006). No association was found between anti-S IgG antibody level and the majority of demographic characteristics after receiving both the 1^st^ (sex p: 0.850) and the 2^nd^ vaccine dose (sex p: 1.00). |
| Maruyama, 2022 [73] | Japan | Prospective cohort study (daily online questionnaires up to 7 days post-vaccination) | Pfizer | 374 healthcare workers (40% males) | The incidence of AEs was higher after the 2^nd^ dose compared to the 1^st^ dose. Females had an increased incidence and numeric rating scores (NRS) for both local and systemic AE, in particular for headache, skin pain, erythema, itching, and muscle pain, with mostly higher values after 2^nd^ vaccine doses than males (p<0.05). No sex-based difference was detected for the incidence of fever after the 1^st^ vaccine dose, whereas the incidence and NRS values of fever tended to be higher in females than in males after the 2^nd^ vaccine dose. The incidence of general fatigue, chills, joint pain and diarrhea was significantly higher among females than males after the 2^nd^ vaccine dose (p<0.05). |
| Mohammed, 2021 [45] | Saudi Arabia | Cross-sectional study (online questionnaire) | Pfizer | 386 vaccinees (46% males) | Female sex was significantly associated with a higher odds of reporting a side effect after both vaccine doses (OR 1.29) |
| Moll, 2022 [46] | Mexico | Cross-sectional study (online questionnaire) | AstraZeneca, Johnson& Johnson, Moderna, Pfizer, Sinovac Life Sciences, Gamaleya’s Sputnik V, CanSinoBIO | 4,024 respondents (20% males) | Female sex, young age (<50 years), and history of allergies, are associated with extent (absent, local, systemic, or both) and severity of adverse effects (AEs). Adjusted OR for females *vs.* males: - Extent dose 1: 1.45, 95%CI 1.25-1.68, p: 0.00001;  - Extent dose 2: 1.27, 95%CI 1.03-1.57, p: 0.025;  - Severity dose 1: 1.52, 95%CI 1.30-1.78, p: 0.0001. |
| Muluneh, 2022 [47] | (Northwest) Ethiopia | Cross-sectional study | AstraZeneca | 314 healthcare workers (71% males) | The risk of developing an AEFI was higher in females compared to males (aOR 2.75, 95%CI 1.15-6.58). |
| Nachtigall, 2022 [86] | Germany | Retrospective cohort study | AstraZeneca, Moderna, Pfizer | 8375 vaccinees (26% males) | The occurrence of both reactogenicity and of incapacity to work differed between sexes, vaccines and between 1^st^ and 2^nd^ vaccination. The male sex was also associated with a lower risk for both, impairment (OR 0.66) and loss of working days (OR 0.72). |
| Namiki, 2022 [13] | Japan | Cross-sectional study | Pfizer | 1681 vaccinees (49% males) | The regression models showed that the total adverse events after the 1^st^ dose were associated with female sex (OR 1.93, p: 1.37×10^-6^). The total adverse events after the 2^nd^ dose of Pfizer were positively associated with female sex (OR 2.19, p: 6.05 × 10^-6^) and negatively with age (OR 0.98, p: 3.25×10^-3^) |
| Nguyen, 2021 [48] | Vietnam | Cross-sectional study (questionnaire) | AstraZeneca | 1082 (34% males) | For almost all symptoms (22/24), percentages of females who reported having symptoms were higher than males. |
| Nishizawa, 2022 [49] | Japan | Cross-sectional study (questionnaire) | Pfizer | 649 healthcare workers who received the 3^rd^ vaccine dose (22% males) | Compared to males, females experienced a higher incidence of several adverse reactions, both systemic and local, such as headache (F 62.6% *vs.* M 48.6%), nausea (F 16.2% *vs.* M 5.0%), myalgia (F 65.0% *vs.* M 50.7%), arthralgia (F 47.6% *vs.* M 25.9%), injection site redness (F 18.7% *vs.* M 9.3%), pruritus (F 16.8% *vs.* M 8.6%) (all p<0.05). |
| Oh, 2022 [87] | South Korea | Retrospective cohort study | Moderna, Pfizer | 683 vaccinated individuals (37.6% males) | Among the 683 patients, the hospital admission rate was higher among males than among females (M 3.9% *vs.* F 1.9%) although there was no significant difference. A total of 70.8% of males and 65.7% of females visited the emergency departments after the 1^st^ vaccine dose, and the number of visits after the 1^st^ vaccine dose were highest (compared to the 2^nd^ dose) both in males and females, except for the 17-19 years age group. Males experienced more frequently chest pain/discomfort than females (M 81.7% *vs.* F 70%; p: 0.003), whereas females suffered the most from dyspnea and palpitation. |
| Otani, 2021 [50] | Japan | Cross-sectional study (questionnaire) and serum anti-SARS-CoV-2 IgG was measured | Pfizer | 338 vaccinees (29% males) | The incidence of symptoms after the 1^st^ (p: 0.026) and 2^nd^ (p: 0.007) doses of the vaccine, and the incidence of itching (p: 0.015), fatigue (p: 0.002), headache (p: 0.026), and rhinorrhea (p: 0.011) after the 1^st^ dose; and itching (p< 0.001), redness (p: 0.038), fatigue (p: 0.015), and headache (p: 0.003) after the 2^nd^ dose, differed significantly by sex. The incidence of adverse reactions after the vaccine was higher in females than in males. |
| Powell, 2021 [88] | England | Retrospective cohort study (questionnaire) | AstraZeneca, Pfizer (heterologous compared to homologous prime-boost COVID-19 vaccination) | 1,313 included in survey, for AstraZeneca/Pfizer (24% males) for Pfizer/AstraZeneca (35% males) for AstraZeneca/ AstraZeneca (35% males) and for Pfizer/Pfizer recipients (28% males). | Reactogenicity was higher in females than males for both doses across immunization schedules |
| Rahmani, 2021 [51] | Italy | Cross-sectional study (online questionnaire) | Pfizer | 275 (45% males) | At the univariate analysis, upon the 1^st^ vaccination dose, associations were found by between being female and an increased reporting of:  - Headache (χ2 8.20, OR 2.06, 95%CI 1.25–3.38, p: 0.004);  - Moderate pain (χ2 4.47, OR 1.79, 95%CI 1.10–2.90, p: 0.035).  After the 2^nd^ dose sex was positively associated with:  - Fatigue (χ2 3.99, OR 1.75, 95%CI 1.01–3.02, p: 0.046);  - Severe headache (χ2 4.75, OR 4.72, 95%CI 1.03–21.7, p: 0.029);  - Muscle/joint pain (χ2 5.53, OR 1.79, 95%CI 1.10–2.90, p: 0.019). |
| Riad, 2021 [53] | Slovakia | Cross-sectional study (questionnaire) | Pfizer | 522 vaccinees (23% males) | Females (88.1%) had a statistically significantly (χ2 7.19; p: 0.007) higher prevalence of local side effects than males (78.3%). Similarly, females (1.07 ± 0.62) had a statistically significantly (U: 21375; p: 0.016) higher intensity of local side effects than males (0.94±0.69). Overall, 70.5% of the vaccinees reported ≥1 systemic side effect, with the female participants (75.6%) being more significantly (χ2 22.07; p<0.001) affected than the male participants (53.3%). Additionally, the intensity of systemic side effects was statistically significantly (U: 18969.5; p<0.001) higher in females (2.29±2.09) than males (1.62±2.00). |
| Riad, 2021 [54] | Czech Republic | Cross-sectional study (questionnaire) | Moderna, Pfizer | 539 vaccinees (30% males) | Females were more likely to experience any side effect and local side effects as compared to males (any side effect aOR: 2.57, 95%CI 1.10-5.97, p: 0.029; local side effect aOR: 2.90, 95%CI 1.47-5.72, p: 0.002). No significant differences were found in the onset of local side effects between females and males. In general, the mean duration of local side effects was significantly different (U: 21,219.5, p: 0.017) between females (2.15±0.96 days) and males (1.93±0.88 days). Female participants (74.6%) had a slightly higher level of systemic side effects prevalence compared to male participants (67.7%). Females (2.48±2.19) had a significantly (U: 25,498.5, p: 0.007) higher level of systemic side effects intensity compared to males (1.94±2.08). |
| Riad, 2021 [55] | Germany and Czech Republic | Cross-sectional study (questionnaire) | AstraZeneca | 92 vaccinees (23% males) | For local side effects, the mean total was higher in females (0.97±0.77) than males (0.86±0.73). For systemic side effects, female participants had an insignificantly higher mean total (3.72±2.34) than the males (3.29±2.22). |
| Ripabelli, 2022 [52] | Italy | Cross-sectional study (questionnaire) | Moderna, Pfizer | 340 individuals (39% males) | Female sex was a risk factor for mild AEFI (ORs 1.86 and 2.82) and common AEFI (ORs 3.45 and 2.15) |
| Rivera-Izquierdo, 2021 [14] | Spain | Prospective cohort study | Pfizer | 3969 (27% males) | Females reported more frequently adverse reactions than males (p: 0.027). After adjusting for the main confounders in the multivariate analysis, female sex, vaccination regimen (single-dose), and antecedent COVID-19 infection remained as the main factors associated with the report of adverse reactions. |
| Rolfes, 2022 [2] | The Netherlands | Prospective cohort study (online questionnaires) | AstraZeneca, Johnson& Johnson, Moderna, Pfizer | 3763 vaccinees who received the Pfizer vaccine (47.1% males), 7962 who received the AstraZeneca vaccine (12.7% males), 935 who received the Johnson& Johnson vaccine (13.7% males) and 1275 who received the Moderna vaccine (21.6% males). | Sex differences were found in the incidence of both overall reactogenicity (M *vs.* F: OR: 0.48, 95%CI 0.45-0.52, p<0.001) and systemic reactogenicity (M *vs.* F: OR 0.52, 95%CI 0.48-0.57, p<0.001). |
| Sa, 2022 [6] | United States | Spontaneous Reporting System for vaccines (VAERS) | Johnson& Johnson, Moderna, Pfizer | 481,172 (28% males) individuals reported adverse events | For common AEs, the risk to males (OR 0.62, 95%CI 0.61–0.63) was lower than to females. For severe AEs, the risk among males (OR 1.37, 95%CI 1.34–1.41) was higher than among females, except for anaphylaxis. |
| Saita, 2022 [74] | Japan | Prospective cohort study (questionnaire) | Pfizer | 3254 vaccinees after 1^st^ dose (34% males), 3165 after 2^nd^ dose (35% males) | Multivariate analysis was performed to assess the differences in reactogenicity by sex and age, for the 1^st^ dose and the 2^nd^ dose. Females were found to have higher rates of reactogenicity in all items, compared to males. |
| Sanyaolu, 2022 [90] | United States | Spontaneous Reporting System for vaccines (VAERS) | Johnson& Johnson, Moderna, Pfizer | 200 evaluated cases, 151 were females, 48 males, and one of unknown sex. | Stratification of sex by the top 10 systemic reactions yielded a significant p-value of 0.002 (p<0.05). Both males and females who received the Pfizer vaccine experienced all five systemic reactions to some degree. Male recipients of the Moderna vaccine seemed to experience fewer systemic side effects than their female counterparts. Both the males and females who received the Johnson&Johnson vaccine seemed to experience fewer systemic reactions. |
| Sauserienė, 2022 [75] | Lithuania | Prospective cohort study (questionnaire) and SARS-CoV-2 IgG/IgM rapid test | Pfizer | 4181 vaccinees (77% female) | AEFIs occurred 1.3 times more frequently in females than males (F 56.6% *vs*. M 43.5%, p<0.001). Females were more likely to refer to a healthcare institution than males (F 2.9% *vs*. M 1.6%, p: 0.02). |
| Shay, 2021 [91] | United States | Spontaneous Reporting System for vaccines (VAERS) and V-safe Intensive Monitoring data | Johnson& Johnson | 13,725 adverse event reports (34% males) and 338,765 in V-safe (40% males) | VAERS data 66.2% of cases about females. Of all non-serious cases 66.6% about females. Of all serious cases 57.1 about females. Half of the cases with a fatal outcome (n=88) were females. V-safe data not stratified by sex. |
| Ughi, 2021 [56] | Italy | Cross-sectional study | Pfizer | 7,014 (49% males) | Sex was significantly associated with reporting of AEFIs in the univariable and multivariable analysis, using males as the reference group. OR univariate analysis: 2.68 (2.32-3.09), p<0.001, OR multivariate analysis: 2.58 (2.22-2.99), p<0.001. |
| Undugodage, 2021 [77] | Sri Lanka | Prospective cohort study (questionnaire [both paper and online]) | AstraZeneca | 4478 (36% males) | Females reported a higher frequency of AEs (93.6%) compared to males (91.1%). All the individual symptoms except feverishness (temperature <38.4°c) were commoner among females than males and the difference was statistically significant (all p<0.05). Severe headache was more common among females (263, 16.6%) than males (92, 13.3%) and the difference was statistically significant (p: 0.002) |
| Urakawa, 2022 [76] | Japan | Prospective cohort study (questionnaire) | Pfizer | 548 (43% males) | For many AEs, females were found to have a significantly higher incidence of AEs than males. Multivariate analysis: injection site reaction and fatigue were significantly more likely to occur in females (OR 1.71 and 1.57, respectively) after the 1^st^ dose. Fatigue, chills, fever, arthralgia, myalgia, and headache were significantly more likely to occur in females (OR 1.74, 2.34, 1.79, 1.88, 1.88, and 2.27, respectively) after the 2^nd^ dose. |
| Uwamino, 2022 [78] | Japan | Prospective cohort study | Pfizer | 646 vaccinees (29% males) | Female sex was significantly associated with a higher frequency of reported AEFIs as compared to male sex. |
| Vigezzi, 2021 [57] | Italy | Cross-sectional study | Pfizer | 2659 hospital personnel (27% males) | Females were more likely to report a non-severe AEFI (RRR 1.65, 95%CI 1.37-1.99, p<0.01) as well as a severe AEFI (RRR 3.33, 95%CI 2.30-4.82, p<0.01) as compared to males. |
| Warkentin, 2022 [79] | Germany | Prospective cohort study (online questionnaires) | AstraZeneca, Moderna, Pfizer | 9146 vaccinees responded to at least one survey (the short-term and/or long-term survey), of which 8145 (43% males) completed the short-term survey, 7104 (40% males) completed the long-term survey. | Among vaccinees who completed the short-term survey, females had an higher odds of experiencing reactogenicity than males (OR 2.23, 95%CI 2.00-2.49). Female sex was positively associated with more frequent medical consultations (based on the long-term survey data), than male sex (in the AstraZeneca/AstraZeneca vaccine group: F 16% *vs*. M 14%; in the AstraZeneca/mRNA vaccine group: F 20% *vs*. M 12%; in the mRNA/mRNA vaccine group: F 19% *vs*. M 12%; OR 1.86, 95%CI 1.62-2.14). Overall, the reactogenicity of the heterologous regimen was found to be higher compared to homologous regimens, regardless of sex stratification. |
| Wi, 2021 [80] | South Korea | Prospective cohort study | AstraZeneca, Pfizer | 1520 healthcare workers (sex distribution not provided) | A slightly higher percentage of males *vs.* females reported an AEFI after the 1^st^ and 2^nd^ dose of Pfizer vaccination (1^st^ dose: M 51.8% *vs.* F 49.0%; 2^nd^ dose: M 59.0% *vs.* F 41.0%). An opposite pattern was observed for the 1^st^ dose of AstraZeneca (M 85.3% *vs.* F 93.0%, p-value: <0.001). |
| Xiong, 2021 [92] | United States | Data from VAERS | Moderna, Pfizer | 8976 vaccinated individuals who reported an adverse event (21% males) | More females than males reported an AE. The top 10 most frequently reported AEs were similar between males and females. More serious, life-threatening AEs, hospitalizations and deaths reported for males compared to females:  - serious AE: aOR 1.50, 95%CI 1.25-1.80;  - death: aOR 3.31, 95%CI 2.28-4.82;  - hospitalization: aOR 1.32, 95%CI 1.08-1.63. |
| Zhang, 2022 [93] | China | Open trial | Pfizer, CoronaVac | 189 vaccinees of which 94 received 2 doses of Pfizer (44% males) and 95 received 2 doses of CoronaVac (36% males) | Females had an increased AE rate following both the Pfizer and CoronaVac doses compared to males. In the Pfizer vaccine group, percentages of females *vs.* males who reported the AE were higher for headache (F 45.7% *vs.* M 29.4%), nausea (F 19.6% *vs.* M 8.8%), joint pain (F 26.1% *vs.* M 14.7%), and diarrhea (F 13.0% *vs.* M 2.9%). In the CoronaVac vaccine group, percentages of females who reported the AE were higher for headache (F 26.5% *vs.* M 0.0%), tiredness (F 38.2% *vs.* M 5.3%), nausea (F 11.8% *vs.* M 0.0%) and muscle pain (F 23.5% *vs.* M 10.5%). Female participants showed higher levels of neutralizing antibodies after receiving either Pfizer or CoronaVac vaccines than males. |
| Zhao, 2021 [94] | United States | Data from VAERS | Moderna, Pfizer | 15785 adverse event reports | 156 AE reports contained urologic symptoms, 54% of these were about female patients. |

AE(s): adverse effect(s). OR: odds ratio. F: female. M: male. RR: risk ratio. AEFI(s): adverse event(s) following immunization. SD: standard deviation. aOR: adjusted odds ratio. VAERS: Vaccine Adverse Event Reporting System. RRR: relative risk ratio. LTAEs: long-term adverse events.
*percentage of males enrolled (if provided) or number of cases & controls.

**References Table S8 (also included in the reference list of the manuscript)**

**2. Rolfes**, L., et al., COVID-19 vaccine reactogenicity–A cohort event monitoring study in the Netherlands using patient reported outcomes. Vaccine, 2022. 40(7): p. 970-976.
**5. Kant**, A., et al., Description of frequencies of reported adverse events following immunization among four different COVID-19 vaccine brands. Drug safety, 2022. 45(4): p. 319-331.
**6. Sa**, S., et al., The safety of mRNA-1273, BNT162b2 and JNJ-78436735 COVID-19 vaccines: safety monitoring for adverse events using real-world data. Vaccines, 2022. 10(2): p. 320.
**12. Green**, M.S., et al., Gender differences in adverse events following the Pfizer-BioNTech COVID-19 vaccine. Vaccines, 2022. 10(2): p. 233.
**13. Namiki**, T., et al., Adverse events after BNT162b2 mRNA COVID-19 vaccination in health care workers and medical students in Japan. Journal of Infection and Chemotherapy, 2022.
**14. Rivera-Izquierdo**, M., et al., Factors Associated with Adverse Reactions to BNT162b2 COVID-19 Vaccine in a Cohort of 3969 Hospital Workers. Vaccines, 2021. 10(1): p. 15.
**17.** **Adam**, M., et al., Evaluation of post-vaccination symptoms of two common COVID-19 vaccines used in Abha, Aseer Region, Kingdom of Saudi Arabia. Patient preference and adherence, 2021. 15: p. 1963.
**18. Al Bahrani**, S., et al., Safety and reactogenicity of the ChAdOx1 (AZD1222) COVID-19 vaccine in Saudi Arabia. International Journal of Infectious Diseases, 2021. 110: p. 359-362**.
19. Al Ghafri**, T.S., et al., Reporting at Least One Adverse Effect Post-COVID-19 Vaccination From Primary Health Care in Muscat. Cureus, 2021. 13(8).
**20. Alessa**, M.Y., et al., The Side Effects of COVID-19 Vaccines and Its Association With ABO Blood Type Among the General Surgeons in Saudi Arabia. Cureus, 2022. 14(3).
**21. Alghamdi**, A.N., et al., BNT162b2 and ChAdOx1 SARS-CoV-2 post-vaccination side-effects among Saudi vaccinees. Frontiers in Medicine, 2021: p. 1796.
**22. Aliberti**, S.M., et al., Gender and AB0 Blood Type Differences in a Unicentric Group of University Professors in Southern Italy Who Received the Vaxzevria COVID-19 Vaccine: A Cross-Sectional Survey of Vaccine Side Effects, Attitudes, and Hesitation. Vaccines, 2022. 10(3): p. 373.
**23. Al Khames Aga**, Q.A., et al., Safety of COVID‐19 vaccines. Journal of medical virology, 2021. 93(12): p. 6588-6594.
**24. Almohaya**, A.M., et al., Early solicited adverse events following the BNT162b2 mRNA vaccination, a population survey from Saudi Arabia. Preventive Medicine Reports, 2021. 24: p. 101595.
**25. Almufty**, H.B., et al., Potential adverse effects of COVID19 vaccines among Iraqi population; a comparison between the three available vaccines in Iraq; a retrospective cross-sectional study. Diabetes & Metabolic Syndrome: Clinical Research & Reviews, 2021. 15(5): p. 102207.
**26. Bauernfeind**, S., et al., Association between reactogenicity and immunogenicity after vaccination with BNT162b2. Vaccines, 2021. 9(10): p. 1089.
**27. Borroni**, E., et al., Side effects among healthcare workers from a large Milan university hospital after second dose of BNT162b2 mRNA COVID-19 vaccine. La Medicina del lavoro, 2021. 112(6): p. 477.
**28. Bukhari**, A.E., et al., Cutaneous adverse reactions to coronavirus vaccines: A Saudi nationwide study. Dermatologic Therapy, 2022: p. e15452.
**29. Canlas**, F.Q., S. Nair, and I.D. Paat, Exploring COVID-19 Vaccine Side Effects: A Correlational Study Using Python. Procedia Computer Science, 2022. 201: p. 752-757.
**30. Cohen**, G., et al., Immunogenicity and reactogenicity after heterologous prime-boost vaccination with CoronaVac and ChAdox1 nCov-19 (AZD1222) vaccines. Human Vaccines & Immunotherapeutics, 2022: p. 1-7.
**31. Costantino**, M., et al., Adverse Events Associated with BNT162b2 and AZD1222 Vaccines in the Real World: Surveillance Report in a Single Italian Vaccine Center. Journal of clinical medicine, 2022. 11(5): p. 1408.
**32. Cuschieri**, S., et al., Adverse reactions to Pfizer‐BioNTech vaccination of healthcare workers at Malta's state hospital. International Journal of Clinical Practice, 2021. 75(10): p. e14605.
**33. Dar-Odeh**, N., et al., Long-term adverse events of three COVID-19 vaccines as reported by vaccinated physicians and dentists, a study from Jordan and Saudi Arabia. Human Vaccines & Immunotherapeutics, 2022. 18(1): p. 2039017.
**34. Elnaem**, M.H., et al., COVID-19 Vaccination Attitudes, Perceptions, and Side Effect Experiences in Malaysia: Do Age, Gender, and Vaccine Type Matter? Vaccines, 2021. 9(10): p. 1156.
**35. El-Shitany**, N.A., et al., Minor to moderate side effects of Pfizer-BioNTech COVID-19 vaccine among Saudi residents: a retrospective cross-sectional study. International journal of general medicine, 2021. 14: p. 1389.
**36. Gan**, L.-L., et al., Adverse events following BNT162b2 mRNA COVID-19 vaccination among healthcare workers: A single-centre experience in Malaysia. MJM, 2022. 77(3): p. 300.
**37. Ganesan**, S., et al., Vaccine Side Effects Following COVID-19 Vaccination Among the Residents of the UAE—An Observational Study. Frontiers in public health, 2022. 10.
**38. Hatmal**, M.m.M., et al., Reported adverse effects and attitudes among Arab populations following COVID-19 vaccination: a large-scale multinational study implementing machine learning tools in predicting post-vaccination adverse effects based on predisposing factors. Vaccines, 2022. 10(3): p. 366.
**39. Kang**, Y.M., et al., Reactogenicity after the first and second doses of BNT162b2 mRNA coronavirus disease vaccine: a single-center study. Clinical and experimental vaccine research, 2021. 10(3): p. 282.
**40. Khan**, M., et al., Tracking Side Effects of the COVID-19 Vaccine in Mymensingh District of Bangladesh. Mymensingh Medical Journal: MMJ, 2022. 31(1): p. 1-9.
**41. Kitagawa**, H., et al., Adverse reactions to the BNT162b2 and mRNA-1273 mRNA COVID-19 vaccines in Japan. Journal of Infection and Chemotherapy, 2022. 28(4): p. 576-581.
**42. Klugar**, M., et al., Side effects of mRNA-based and viral vector-based COVID-19 vaccines among German healthcare workers. Biology, 2021. 10(8): p. 752.
**43. Lounis**, M., et al., Side Effects of COVID-19 Inactivated Virus vs. Adenoviral Vector Vaccines: Experience of Algerian Healthcare Workers. Frontiers in Public Health, 2022. 10.
**44. Mahallawi**, W.H. and W.A. Mumena, Reactogenicity and Immunogenicity of the Pfizer and AstraZeneca COVID-19 Vaccines. Frontiers in Immunology, 2021: p. 5169.
**45. Mohammed**, R.A., et al., A survey on the side effects of Pfizer/BioNTech COVID-19 vaccine among vaccinated adults in Saudi Arabia. Cureus, 2021. 13(11).
**46. Moll**, M.E.C., et al., Extension and Severity of Self-Reported Side Effects of Seven COVID-19 Vaccines in Mexican Population. Frontiers in public health, 2022. 10.
**47. Muluneh**, A.G., et al., More Than Three-Fourths of AstraZeneca (ChAdox1 COV-19) COVID-19 Vaccinated Individuals Develop Post Immunization Adverse Event in Northwest Ethiopia. Infection and Drug Resistance, 2022. 15: p. 2409.
**48. Nguyen**, H.A., et al., Factors influencing adverse events following immunization with AZD1222 in Vietnamese adults during first half of 2021. Vaccine, 2021. 39(44): p. 6485-6491.
**49. Nishizawa**, T., et al., Adverse reactions of BNT162b2 vaccine booster against COVID‐19 in Japan. Journal of General and Family Medicine, 2022.
**50. Otani**, J., R. Ohta, and C. Sano, Association between immunoglobulin G levels and adverse effects following vaccination with the BNT162b2 vaccine among Japanese healthcare workers. Vaccines, 2021. 9(10): p. 1149.
**51. Rahmani**, A., et al., Reactogenicity of bnt162b2 mrna covid-19 vaccine in a young working age population: A survey among medical school residents, within a mass vaccination campaign, in a regional reference teaching hospital in italy. Vaccines, 2021. 9(11): p. 1269.
**52. Ripabelli**, G., et al., Active surveillance of adverse events in healthcare workers recipients after vaccination with COVID-19 BNT162b2 vaccine (Pfizer-BioNTech, Comirnaty): a cross-sectional study. Journal of Community Health, 2022. 47(2): p. 211-225.
**53. Riad**, A., et al., Side effects of mRNA-based COVID-19 vaccine: nationwide phase IV study among healthcare workers in Slovakia. Pharmaceuticals, 2021. 14(9): p. 873.
**54. Riad**, A., et al., Side effects of mRNA-based COVID-19 vaccines among young adults (18–30 years old): an independent post-marketing study. Pharmaceuticals, 2021. 14(10): p. 1049.
**55. Riad**, A., et al., Safety of ChAdOx1 nCoV-19 vaccine: independent evidence from two EU states. Vaccines, 2021. 9(6): p. 673.
**56. Ughi**, N., et al., Host factors and history of SARS-CoV-2 infection impact the reactogenicity of BNT162b2 mRNA vaccine: results from a cross-sectional survey on 7,014 workers in healthcare. European Review for Medical and Pharmacological Sciences, 2021. 25(24): p. 7985-7996.
**57. Vigezzi**, G.P., et al., Safety surveillance after BNT162b2 mRNA COVID-19 vaccination: results from a cross-sectional survey among staff of a large Italian teaching hospital. Acta Bio Medica: Atenei Parmensis, 2021. 92(Suppl 6).
**58. Alharbi**, N.K., et al., Outcomes of single dose COVID-19 vaccines: Eight month follow-up of a large cohort in Saudi Arabia. Journal of infection and public health, 2022. 15(5): p. 573-577.
**59. Amodio**, E., et al., Adverse Reactions to Anti-SARS-CoV-2 Vaccine: A Prospective Cohort Study Based on an Active Surveillance System. Vaccines, 2022. 10(3): p. 345.
**60. Anastassopoulou**, C., et al., Age and sex associations of SARS-CoV-2 antibody responses post BNT162b2 vaccination in healthcare workers: A mixed effects model across two vaccination periods. PloS one, 2022. 17(4): p. e0266958.
**61. Azzolini**, E., et al., Short-Term Adverse Events and Antibody Response to the BNT162b2 SARS-CoV-2 Vaccine in 4156 Health Care Professionals. Vaccines, 2022. 10(3): p. 439.
**62. Bae**, S., et al., Adverse reactions following the first dose of ChAdOx1 nCoV-19 vaccine and BNT162b2 vaccine for healthcare workers in South Korea. Journal of Korean medical science, 2021. 36(17).
**63. Beatty**, A.L., et al., Analysis of COVID-19 vaccine type and adverse effects following vaccination. JAMA network open, 2021. 4(12): p. e2140364-e2140364.
**64. Chapin-Bardales**, J., et al., Reactogenicity within 2 weeks after mRNA COVID-19 vaccines: Findings from the CDC v-safe surveillance system. Vaccine, 2021. 39(48): p. 7066-7073.
**65. Coggins**, S.A.A., et al. Adverse effects and antibody titers in response to the BNT162b2 mRNA COVID-19 vaccine in a prospective study of healthcare workers. in Open forum infectious diseases. 2022. Oxford University Press US.
**66. Darraj**, M.A. and H.M. Al-Mekhlafi, Prospective Evaluation of Side-Effects Following the First Dose of Oxford/AstraZeneca COVID-19 Vaccine among Healthcare Workers in Saudi Arabia. Vaccines, 2022. 10(2): p. 223.
**67. Filippatos**, F., et al., Association of clinical and epidemiological characteristics with COVID-19 BNT162b2 mRNA vaccine short-term adverse reactions in healthcare workers. Human Vaccines & Immunotherapeutics, 2021. 17(12): p. 4755-4760.
**68. Izumo**, T., et al., Side effects and antibody titer transition of the BNT162b2 messenger ribonucleic acid coronavirus disease 2019 vaccine in Japan. Respiratory Investigation, 2021. 59(5): p. 635-642.
**69. Jacobson**, M.A., et al., Incidence and Characteristics of Delayed Injection Site Reaction to the mRNA-1273 Severe Acute Respiratory Syndrome Coronavirus 2 (SARS-CoV-2) Vaccine (Moderna) in a Cohort of Hospital Employees. Clinical Infectious Diseases, 2022. 74(4): p. 591-596.
**70. Koh**, J.S., et al., Hospital-based observational study of neurological disorders in patients recently vaccinated with COVID-19 mRNA vaccines. Journal of the Neurological Sciences, 2021. 430: p. 120030.
**71. Lee**, S.W., et al., Risk Factors for Grade 3 to Grade 4 Adverse Reactions to the ChAdOx1 nCoV-19 Vaccine (AZD1222) Against SARS-CoV-2. Frontiers in medicine, 2021. 8.
**72. Lee**, Y.W., et al., Adverse reactions of the second dose of the BNT162b2 mRNA COVID-19 vaccine in healthcare workers in Korea. Journal of Korean medical science, 2021. 36(21).
**73. Maruyama**, A., et al., Adverse reactions to the first and second doses of Pfizer-BioNTech COVID-19 vaccine among healthcare workers. Journal of Infection and Chemotherapy, 2022. 28(7): p. 934-942.
**74. Saita**, M., et al., Reactogenicity following two doses of the BNT162b2 mRNA COVID-19 vaccine: real-world evidence from healthcare workers in Japan. Journal of Infection and Chemotherapy, 2022. 28(1): p. 116-119.
**75. Sauserienė**, J., et al., Adverse events and immunogenicity of mRNA-Based COVID-19 vaccine among healthcare workers: a single-centre experience. Medicina, 2022. 58(3): p. 441.
**76. Urakawa**, R., et al., Impact of age, sex and medical history on adverse reactions to the first and second dose of BNT162b2 mRNA COVID-19 vaccine in Japan: a cross-sectional study. BMC Infectious Diseases, 2022. 22(1): p. 1-8.
**77. Undugodage**, C., et al., Reactogenicity to ChAdOx1 nCoV-19 vaccine in health care workers: A multicenter observational study in Sri Lanka. The Ceylon Medical Journal, 2021. 66(4): p. 177-184.
**78. Uwamino**, Y., et al., Young age, female sex, and presence of systemic adverse reactions are associated with high post-vaccination antibody titer after two doses of BNT162b2 mRNA SARS-CoV-2 vaccination: An observational study of 646 Japanese healthcare workers and university staff. Vaccine, 2022. 40(7): p. 1019-1025.
**79. Warkentin**, L., et al., Reactogenicity after heterologous and homologous COVID-19 prime-boost vaccination regimens: descriptive interim results of a comparative observational cohort study. BMC Infectious Diseases, 2022. 22(1): p. 1-15.
**80. Wi**, Y.-M., S.-H. Kim, and K.-R. Peck, Early adverse events between mRNA and adenovirus-vectored COVID-19 vaccines in healthcare workers. Vaccines, 2021. 9(8): p. 931.
**81. Almohaya**, A.M., et al., Acute unsolicited adverse events following BNT162b2 vaccine in Saudi Arabia, a real-world data. Vaccine, 2022. 40(3): p. 477-482.
**82. Hibino**, M., et al. Delayed injection site reaction after mRNA-1273 vaccination in Japan: a retrospective, cross-sectional study. in Open Forum Infectious Diseases. 2021. Oxford University Press US.
**83. Higashino**, T., et al., Assessment of Delayed Large Local Reactions After the First Dose of the SARS-CoV-2 mRNA-1273 Vaccine in Japan. JAMA dermatology, 2022.
**84. Hoffmann**, M.A., et al., Age-and sex-graded data evaluation of vaccination reactions after initial injection of the BNT162b2 mRNA vaccine in a local vaccination center in Germany. Vaccines, 2021. 9(8): p. 911.
**85. Loosen**, S.H., et al., Factors Associated with Non-Severe Adverse Reactions after Vaccination against SARS-CoV-2: A Cohort Study of 908,869 Outpatient Vaccinations in Germany. Vaccines, 2022. 10(4): p. 566.
**86. Nachtigall**, I., et al., Effect of gender, age and vaccine on reactogenicity and incapacity to work after COVID-19 vaccination: a survey among health care workers. BMC Infectious Diseases, 2022. 22(1): p. 1-13.
**87. Oh**, T.H., et al., Clinical features of patients presenting to the emergency department with cardiovascular adverse reactions after COVID-19 mRNA vaccination. Journal of Korean medical science, 2022. 37(9).
**88. Powell**, A.A., et al., Real-world data shows increased reactogenicity in adults after heterologous compared to homologous prime-boost COVID-19 vaccination, March− June 2021, England. Eurosurveillance, 2021. 26(28): p. 2100634.
**89. Kaur**, R.J., et al., Cardiovascular adverse events reported from COVID-19 vaccines: a study based on WHO database. International journal of general medicine, 2021. 14: p. 3909.
**90. Sanyaolu**, A., et al., Reactogenicity to COVID-19 vaccination in the United States of America. Clinical and Experimental Vaccine Research, 2022. 11(1): p. 104.
**91. Shay**, D.K., Safety monitoring of the Janssen (Johnson & Johnson) COVID-19 vaccine—United States, March–April 2021. MMWR. Morbidity and mortality weekly report, 2021. 70.
**92. Xiong**, X., et al., Age and gender disparities in adverse events following COVID-19 vaccination: Real-world evidence based on big data for risk management. Frontiers in medicine, 2021. 8.
**93. Zhang**, R., et al., Correlation of Immunogenicity and Reactogenicity of BNT162b2 and CoronaVac SARS-CoV-2 Vaccines. Msphere, 2022. 7(2): p. e00915-21.
**94. Zhao**, H., et al., Low rates of urologic side effects following coronavirus disease vaccination: an analysis of the food and drug administration vaccine adverse event reporting system. Urology, 2021. 153: p. 11-13.
